# Noise suppression in stochastic genetic circuits using PID controllers

**DOI:** 10.1371/journal.pcbi.1009249

**Published:** 2021-07-28

**Authors:** Saurabh Modi, Supravat Dey, Abhyudai Singh

**Affiliations:** 1 Department of Biomedical Engineering, University of Delaware, Newark, Delaware, United States of America; 2 Department of Electrical and Computer Engineering, University of Delaware, Newark, Delaware, United States of America; University of Pittsburgh, UNITED STATES

## Abstract

Inside individual cells, protein population counts are subject to molecular noise due to low copy numbers and the inherent probabilistic nature of biochemical processes. We investigate the effectiveness of proportional, integral and derivative (PID) based feedback controllers to suppress protein count fluctuations originating from two noise sources: bursty expression of the protein, and external disturbance in protein synthesis. Designs of biochemical reactions that function as PID controllers are discussed, with particular focus on individual controllers separately, and the corresponding closed-loop system is analyzed for stochastic controller realizations. Our results show that proportional controllers are effective in buffering protein copy number fluctuations from both noise sources, but this noise suppression comes at the cost of reduced static sensitivity of the output to the input signal. In contrast, integral feedback has no effect on the protein noise level from stochastic expression, but significantly minimizes the impact of external disturbances, particularly when the disturbance comes at low frequencies. Counter-intuitively, integral feedback is found to amplify external disturbances at intermediate frequencies. Next, we discuss the design of a coupled feedforward-feedback biochemical circuit that approximately functions as a derivate controller. Analysis using both analytical methods and Monte Carlo simulations reveals that this derivative controller effectively buffers output fluctuations from bursty stochastic expression, while maintaining the static input-output sensitivity of the open-loop system. In summary, this study provides a systematic stochastic analysis of biochemical controllers, and paves the way for their synthetic design and implementation to minimize deleterious fluctuations in gene product levels.

## Author summary

In the noisy cellular environment, biochemical species such as genes, RNAs and proteins that often occur at low molecular counts, are subject to considerable stochastic fluctuations in copy numbers over time. How cellular biochemical processes function reliably in the face of such randomness is an intriguing fundamental problem. Increasing evidence suggests that random fluctuations (noise) in protein copy numbers play important functional roles, such as driving genetically identical cells to different cell fates. Moreover, many disease states have been attributed to elevated noise levels in specific proteins. Here we systematically investigate design of biochemical systems that function as proportional, integral and derivative-based feedback controllers to suppress protein count fluctuations arising from bursty expression of the protein and external disturbance in protein synthesis. Our results show that different controllers are effective in buffering different noise components, and identify ranges of feedback gain for minimizing deleterious fluctuations in protein levels.

## 1 Introduction

Advances in single-cell technologies over the last decade have revealed striking differences between individual cells of the same population. For example, the level of a given protein can vary considerably across cells within a population, in spite of the fact that cells are identical clones of each other and are exposed to the same environment [1–6]. Such intercellular stochastic differences in gene expression patterns can have tremendous consequences for biology and medicine [7–12], including stochastic cell-fate assignment [13–19], microbial bet hedging [20–24], bacterial and cancer drug-resistance [25, 26].

Stochastic variations in the intracellular level of protein primarily arise from two main sources:

- Low-copy number fluctuations in underlying biomolecular components (genes, mRNA, proteins). Moreover, this shot noise, usually originating from a simple Poisson birth-death process, is amplified by the fact that transcription of genes is not a continuous process but happens in sporadic bursts [27–31].
- External disturbances in the protein synthesis rate due to fluctuations in expression-related machinery (RNA polymerases, ribosomes, etc.) or intercellular differences in cell-cycle stage/cell size [32–35].

Given these noise sources, cells encode diverse regulatory mechanisms to suppress stochasticity in the level of a protein around a set point. Perhaps the simplest example of this is a negative feedback loop, where the protein directly or indirectly inhibits its own synthesis [36–47]. Such naturally occurring feedbacks have been shown to be key motifs in gene regulatory networks [48]. Furthermore, design of in-vitro/in-silico synthetic feedback systems based on linear or nonlinear Proportional, Integral, and Derivative (PID) controllers is an intense area of current research [49–55].

PID controllers are widely used in industry for maintaining plant output to a desired value against perturbations. Classical control theory states that in the PID control, the control effort or magnitude of the feedback regulation is either proportional to the error (difference between set point and current protein level), integral of error or temporal derivative of error for P, I, D controllers respectively. While the proportional control affects immediate regulation, it suffers from steady state error offset. The integral control removes such an offset but is vulnerable to high frequency disturbances. These disturbances are effectively buffered by derivative control, hence each component serves a purpose. Several biochemical designs of integral feedback controllers have been experimentally implemented in bacterial cells for perfect adaptation in response to environmental perturbations [56, 57]. Naturally existing circuits implementing integral feedback play a key role in regulating cellular heat shock responses [58, 59], and bacterial chemotaxis [60, 61]. Proportional control is synthetically implemented in experiments in conjunction with integral controllers [49]. While derivative control biochemical designs have been proposed [62], their experimental implementation is not yet realized. An effective synthetic experimental implementation of these components requires carefully tuned control parameters. The novel contribution of our work is to provide simple closed form expressions for noise and optimal parameters.

**Fig 1:**
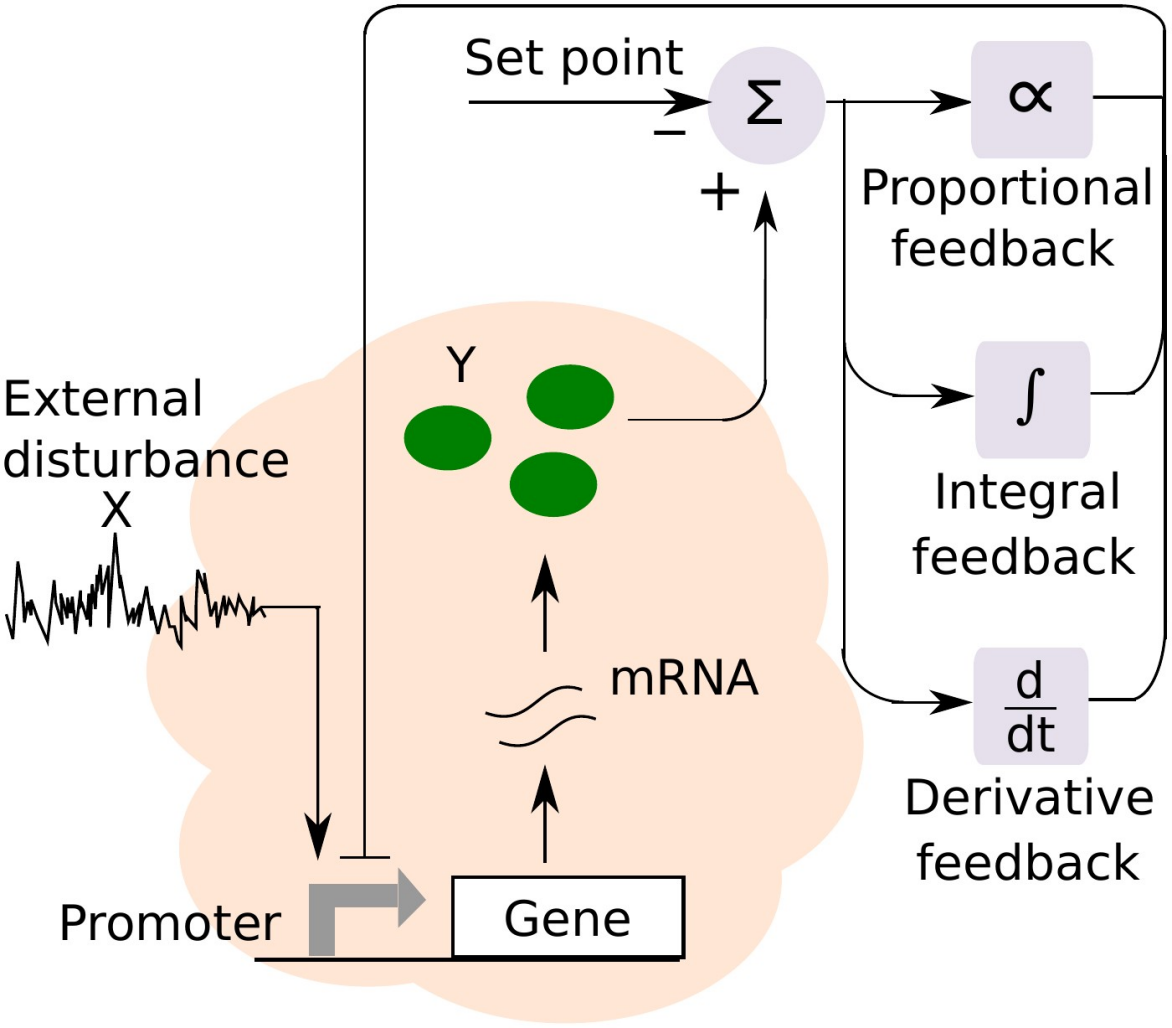
Schematic of the gene expression process, where a gene is transcribed to produce mRNAs. Each mRNA is subsequently translated to synthesize protein *Y* molecules. The expression process is impacted by an upstream external disturbance. Proportional-integral-derivative (PID) controllers can be designed to minimize fluctuations in *Y* copy number around a desired set point.

In Section 2.1, we introduce an open-loop model of stochastic gene expression where the protein is expressed in random bursts, and its expression is impacted by an upstream noisy input (Fig 1). We provide exact analytical formulas for the protein mean and noise levels in open loop. Section 2.1 also introduces the mathematical tools to be used throughout the paper for the analysis of stochastic dynamical systems. In Section 2.2, 2.3 and 2.4, we discuss designs of nonlinear biochemical circuits that function as approximate proportional, integral and derivate controllers, respectively. Given the nonlinearities introduced by feedback loops, we use the linear noise approximation method [63, 64] to investigate their noise suppression properties, and validate the results by performing exact stochastic simulations of the feedback system.

## 2 Results

*Symbols and Notation*: Throughout the paper we denote chemical species by capital letters, and use corresponding small letters for molecular counts. For example, if *Y* denotes a protein species, then *y*(*t*) is the number of molecules of *Y* at time *t* inside the cell. We use angular brackets to denote the expected value of random variables and stochastic processes. Given a scalar random process *y*(*t*) ∈ {0, 1, 2, …} that takes non-negative integer values, then

$$\begin{array}{c} \left\langle y{\left(t\right)}^{m}\right\rangle ≔\sum_{i=0}^{\infty }i^{m}P\left(y\left(t\right)=i\right),\;\;m\in \left\{1,2,\ldots \right\} \end{array}$$ (1)

represent its *m*^*th*^ order uncentered moment and P(y(t)=i) is the probability of having *i* molecules. Steady-state statistical moments are denoted by

$$\begin{array}{c} \bar{\left\langle y^{m}\right\rangle }≔\underset{t\to \infty }{\text{lim}}\left\langle y{\left(t\right)}^{m}\right\rangle . \end{array}$$ (2)

Finally, noise in the level of protein species is quantified by the steady-state coefficient of variation squared (variance divided by mean squared) that is defined as

$$\begin{array}{c} CV_{Y}^{2}≔\frac{\bar{\left\langle y^{2}\right\rangle }-{\bar{\left\langle y\right\rangle }}^{2}}{{\bar{\left\langle y\right\rangle }}^{2}}. \end{array}$$ (3)

### 2.1 Systems modeling of gene expression

We start by introducing simple models of the gene expression process with a particular focus on incorporating noise mechanisms that drive fluctuations in the level of a protein.

#### 2.1.1 Incorporating bursty dynamics

Usually in eukaryotes, transcription of individual genes inside single cells has been shown to occur in bursts of activity, followed by periods of silence [62, 65–69]. Each burst corresponds to the gene stochastically switching to a transcriptionally active state, and then becoming inactive after synthesizing a few messenger RNA (mRNA) transcripts. Such bursts in mRNA can result in the burst of protein copy number as well. In prokaryotes, however, the burst mechanism could be different. The mRNAs are typically unstable with short half-lives compared to proteins they encode, and consequently each mRNA decays after translating a burst of few protein molecules [70, 71]. The combined effect of both these processes (single gene making multiple mRNAs, single mRNA making multiple proteins) is to create a net burst of protein molecules, every time the gene becomes active. Motivated by these experimental findings, we phenomenologically model protein copy-number fluctuations via a *bursty birth-death process* [72–76]. More specifically, bursts arrive at a constant Poisson rate *k*_*y*_ that corresponds to the frequency with which the gene becomes active. Each burst arrival event, results in the synthesis of *B*_*y*_ ∈ {1, 2, …} protein molecules, where the burst size *B*_*y*_ is an independent and identically distributed random variable that is drawn from an arbitrary positively-valued probability distribution.

Let *y*(*t*) denote the intracellular copy number of protein *Y* at time *t*. Then, based on the above model description, the probability of a burst event of size *B*_*y*_ = *j* molecules occurring in the next infinitesimal time interval (*t*, *t* + *dt*] is

$$\begin{array}{c} P\left(y\left(t+dt\right)=y\left(t\right)+j|y\left(t\right)\right)=k_{y}P\left(B_{y}=j\right)dt. \end{array}$$ (4)

Assuming each protein molecule decays with a constant rate *γ*_*y*_, defines the probability for the protein death event occurring in the time interval (*t*, *t* + *dt*] as

$$\begin{array}{c} P\left(y\left(t+dt\right)=y\left(t\right)-1|y\left(t\right)\right)=\gamma_{y}ydt. \end{array}$$ (5)

Having defined an integer-valued continuous-time Markov process *y*(*t*) via the probabilities (4) and (5), we now focus our attention on its statistical moments. We refer the reader to [77–80] for a thorough analysis of moment dynamics for stochastic systems of the form (4) and (5), and only provide the main result here—the time evolution of the expected value of *y*(*t*)^*m*^ is given by

$$\begin{array}{cc} & \frac{d\langle y{\left(t\right)}^{m}\rangle }{dt}=\left\langle G\left(y\right)\right\rangle ,\;m\in \left\{1,2,\ldots \right\} \end{array}$$ (6)

where the infinitesimal generator *G* takes the form

$$\begin{array}{cc} & G\left(y\right)≔\sum_{j=0}^{\infty }k_{y}P\left(B_{y}=j\right)\left[{\left(y+j\right)}^{m}-y^{m}\right]+\gamma_{y}y\left[{\left(y-1\right)}^{m}-y^{m}\right]. \end{array}$$ (7)

Intuitively, the right-hand-side of (7) is simply the product of the change in *y*^*m*^ when an event occurs and the probabilistic rate at which it occurs, summed across all possible events. Substituting the appropriate value of *m* in (6) yields the following moment dynamics

$$\begin{array}{cc} & \frac{d\langle y\rangle }{dt}=k_{y}\left\langle B_{y}\right\rangle -\gamma_{y}\left\langle y\right\rangle \end{array}$$ (8a)

$$\begin{array}{cc} & \frac{d\langle y^{2}\rangle }{dt}=\gamma_{y}\left(\left\langle y\right\rangle -2\left\langle y^{2}\right\rangle \right)+k_{y}\left\langle B_{y}^{2}\right\rangle +2k_{y}\left\langle y\right\rangle \left\langle B_{y}\right\rangle \end{array}$$ (8b)

where 〈*B*_*y*_〉 is the mean protein burst size, and $\langle B_{y}^{2}\rangle$ is its second-order moment. Subsequent steady-analysis of (8) reveals the protein mean and noise levels to be

$$\begin{array}{cc} & \bar{\left\langle y\right\rangle }=\frac{k_{y}\left\langle B_{y}\right\rangle }{\gamma_{y}},\;\;CV_{Y}^{2}=\frac{\left\langle B_{y}\right\rangle +\left\langle B_{y}^{2}\right\rangle }{2\left\langle B_{y}\right\rangle \bar{\left\langle y\right\rangle }}, \end{array}$$ (9)

respectively. *B*_*y*_ = 1 with probability one leads to Poissonian fluctuations in *Y* copy numbers with $CV_{Y}^{2}=1/\bar{\langle y\rangle }$. If the burst size *B*_*y*_ is assumed to be a geometrically-distributed random variable with mean burst size 〈*B*_*y*_〉 (as shown experimentally for an *E. coli* gene [81]), then $\langle B_{y}^{2}\rangle =2{\langle B_{y}\rangle }^{2}-\langle B_{y}\rangle$, and the above noise levels reduce to

$$\begin{array}{c} CV_{Y}^{2}=\frac{\left\langle B_{y}\right\rangle }{\bar{\left\langle y\right\rangle }}=\frac{\gamma_{y}}{k_{y}}. \end{array}$$ (10)

A key point worth mentioning is that the product $CV_{Y}^{2}\times \bar{\langle y\rangle }$ is independent of the burst frequency *k*_*y*_, while $CV_{Y}^{2}$ in (10) is independent of the mean burst size 〈*B*_*y*_〉. Thus, simultaneous measurements of both the mean and protein noise levels allows for discerning whether a change in $\bar{\langle y\rangle }$ is a result of alterations in *k*_*y*_ or 〈*B*_*y*_〉. Interestingly, this noise-based method works quite well in practice, and has successfully elucidated the bursty kinetics of several genes [82–85]. We note that the analytical expressions for the noise obtained in this paper for various controllers are valid for arbitrary burst size distributions. For simulations, the burst size distribution is assumed to be geometric.

#### 2.1.2 Incorporating external disturbance

Next, we introduce another important source of stochasticity that arises from external disturbances in the protein synthesis rate. These disturbances correspond to fluctuations in the abundance of enzymes, such as, transcription factors, RNA polymerases, etc. We lump these factors into a single species *X* and model its stochastic dynamics via a bursty birth-death process [86], analogous to (4) and (5):

$$\begin{array}{cc} & P\left(x\left(t+dt\right)=x\left(t\right)+j|x\left(t\right)\right)=k_{x}P\left(B_{x}=j\right)dt, \end{array}$$ (11a)

$$\begin{array}{cc} & P\left(x\left(t+dt\right)=x\left(t\right)-1|x\left(t\right)\right)=\gamma_{x}xdt. \end{array}$$ (11b)

Here *k*_*x*_ is the Poisson arrival rate of bursts in *X*, *B*_*x*_ is the burst size, and *γ*_*x*_ is the decay rate of *X*. Then, as per (9)

$$\begin{array}{cc} & \bar{\left\langle x\right\rangle }=\frac{k_{x}\left\langle B_{x}\right\rangle }{\gamma_{x}},\;\;CV_{X}^{2}=\frac{\left\langle B_{x}\right\rangle +\left\langle B_{x}^{2}\right\rangle }{2\left\langle B_{x}\right\rangle \bar{\left\langle x\right\rangle }}. \end{array}$$ (12)

The time-scale for the above bursty dynamics of extrinsic factor is $\gamma_{x}^{-1}$ [86]. Therefore, one can independently vary 〈*B*_*x*_〉 and *γ*_*x*_ to change the amplitude and time-scale of the external disturbance. We note, alternatively, the Ornstein-Uhlenbeck (OU) process is also used to model extrinsic noise in the literature [87, 88].

The disturbance can be connected to the synthesis of *Y* by redefining the frequency of protein *Y* bursts to be proportional to *x*(*t*), that is, by replacing *k*_*y*_ with $k_{y}x\left(t\right)/\bar{\langle x\rangle }$. This redefinition includes the division by $\bar{\langle x\rangle }$ to ensure that the average burst arrival rate is *k*_*y*_. While the external disturbance can be incorporated in the burst size, in this paper we consider modulation of burst frequency [89]. This leads to a system of coupled bursty birth-death processes given by (11) and

$$\begin{array}{cc} & P\left(y\left(t+dt\right)=y\left(t\right)+j|y\left(t\right),x\left(t\right)\right)=\frac{k_{y}x}{\bar{\left\langle x\right\rangle }}P\left(B_{y}=j\right)dt \end{array}$$ (13a)

$$\begin{array}{cc} & P\left(y\left(t+dt\right)=y\left(t\right)-1|y\left(t\right),x\left(t\right)\right)=\gamma_{y}ydt \end{array}$$ (13b)

which replaces (4) and (5). The statistical moments of this joint process evolve as per

$$\begin{array}{c} \frac{d\langle y{\left(t\right)}^{m_{1}}x{\left(t\right)}^{m_{2}}\rangle }{dt}=\left\langle G\left(y,x\right)\right\rangle ,\;m_{1},m_{2}\in \left\{0,1,2,\ldots \right\}\\ G\left(y,x\right)≔\sum_{j=0}^{\infty }\frac{k_{y}x}{\bar{\left\langle x\right\rangle }}P\left(B_{y}=j\right)\left[{\left(y+j\right)}^{m_{1}}x^{m_{2}}-y^{m_{1}}x^{m_{2}}\right]\\ +\sum_{j=0}^{\infty }k_{x}P\left(B_{x}=j\right)\left[y^{m_{1}}{\left(x+j\right)}^{m_{2}}-y^{m_{1}}x^{m_{2}}\right]\\ +\gamma_{x}x\left[y^{m_{1}}{\left(x-1\right)}^{m_{2}}-y^{m_{1}}x^{m_{2}}\right]+\gamma_{y}y\left[{\left(y-1\right)}^{m_{1}}x^{m_{2}}-y^{m_{1}}x^{m_{2}}\right] \end{array}$$ (14)

[77–80]. To write moment dynamics in a compact form we define a vector

$$\begin{array}{c} \mu ={\left[\left\langle x\right\rangle ,\left\langle y\right\rangle ,\left\langle xy\right\rangle ,\left\langle x^{2}\right\rangle ,\left\langle y^{2}\right\rangle \right]}^{T} \end{array}$$ (15)

that consists of all the first and second order moments of *x*(*t*) and *y*(*t*). Then, its time evolution is given by a system of linear differential equations

$$\begin{array}{c} \dot{\mu }=\hat{a}+A\mu , \end{array}$$ (16)

where vector $\hat{a}$ and matrix *A* are obtained via (15) by choosing appropriate values of *m*_1_, *m*_2_. Steady-state analysis of (16) results in the same mean *Y* level as (9), and the following noise level

$$\begin{array}{cc} CV_{Y}^{2}= & \overset{\text{Intrinsic}\;\text{noise}}{\overset{︷}{CV_{int}^{2}}}+\overset{\text{External}\;\text{disturbance}}{\overset{︷}{\frac{\gamma_{y}}{\left(\gamma_{y}+\gamma_{x}\right)}CV_{X}^{2},}}\;\;CV_{int}^{2}=\frac{\left\langle B_{y}\right\rangle +\left\langle B_{y}^{2}\right\rangle }{2\left\langle B_{y}\right\rangle \bar{\left\langle y\right\rangle }}, \end{array}$$ (17)

that can be decomposed into two components. The first component $CV_{int}^{2}$ is the noise contribution from stochastic bursts computed earlier in (9), and has been referred to in literature as the *intrinsic noise* in *Y* [90–94]. The second component is the noise contribution of the external disturbance, and has been referred to as the *extrinsic noise* in *Y*. Note that the ratio *γ*_*y*_/(*γ*_*y*_ + *γ*_*x*_) quantifies the time-averaging of upstream fluctuation in *X* by *Y*. For example, fast fluctuations in *X* are efficiently averaged out by *Y*, and this ratio approaches zero for *γ*_*x*_ → ∞. In contrast, slow fluctuations in *X* lead to inefficient time-averaging that increases *Y* noise levels to

$$\begin{array}{c} CV_{Y}^{2}=\;CV_{int}^{2}+CV_{X}^{2},\;\;\;\gamma_{x}\ll \gamma_{y}. \end{array}$$ (18)

Our results will remain the same, even if we model the external disturbance as an OU process instead of the bursty process (See S1 Text section V). Next, we investigate how negative feedback regulation suppresses different noise components in (17) to minimize fluctuations in *Y* copy numbers around it mean $\bar{\langle y\rangle }$.

### 2.2 Noise suppression using proportional controller

**Fig 2:**
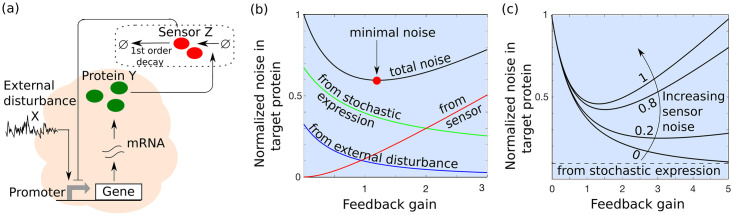
Implementation and noise decomposition for a proportional feedback controller. (a) Schematic of a proportional controller where the protein *Y* is sensed by a noisy sensor *Z* that inhibits the synthesis of *Y*. (b) Different components in the noise levels of protein *Y* from (30) plotted as a function of the feedback gain *f*_*p*_. While feedback selectively attenuates noise due to external disturbance and stochastic expression of *Y*, it amplifies the sensor noise, leading to a non-monotonic profile for the total noise. The noise contribution from the external disturbance decreases rapidly as a function of *f*_*p*_ and approaches zero for *f*_*p*_ → ∞. In contrast, the intrinsic noise decreases slowly and asymptotically approaches a non-zero limit. In this plot, noise levels are normalized to the open-loop noise (*f*_*p*_ = 0), with other parameters chosen as $CV_{Z}^{2}=0.4$, $CV_{int}^{2}=CV_{X}^{2}=0.2$, *γ*_*z*_ = 5*γ*_*y*_ = 15*γ*_*x*_. (c) The normalized total noise in *Y* from (30) with respect to the feedback gain *f*_*p*_ for different levels of sensor noise. The total noise $CV_{Y}^{2}$ is minimized at an optimal feedback gain, which critically depends on the extent of sensor noise $CV_{Z}^{2}$.

To implement a negative feedback loop, we first introduce a new protein species *Z* that functions as a noisy sensor of *Y*. Protein *Z* is also assumed to be synthesized in bursts of size *B*_*z*_, and senses *Y* via its burst frequency *k*_*z*_*y*(*t*) that responds linearly to any changes in *Y* levels. This leads to the following bursty birth-death process for *z*(*t*)

$$\begin{array}{cc} & P\left(z\left(t+dt\right)=z\left(t\right)+j|y\left(t\right),z\left(t\right)\right)=k_{z}yP\left(B_{z}=j\right)dt, \end{array}$$ (19a)

$$\begin{array}{cc} & P\left(z\left(t+dt\right)=z\left(t\right)-1|y\left(t\right),z\left(t\right)\right)=\gamma_{z}zdt, \end{array}$$ (19b)

where *γ*_*z*_ is the decay rate of protein *Z*. Recall from Section 2.1.2 that the frequency of bursts in the *Y* protein was $k_{y}x\left(t\right)/\bar{\langle x\rangle }$ in the open-loop system. To close the feedback loop, we now modify this burst frequency to $k_{y}g\left(z\right)x\left(t\right)/\bar{\langle x\rangle }$, where *g*(*z*) is a positively-valued monotonically decreasing function of *z*(*t*). Typically, *g* takes the form of a Hill function that mechanistically arises from the fast binding-unbinding of the protein to the gene’s promoter region to regulate transcriptional activity [95]. Within this feedback there are three noise mechanisms at play: external disturbance *X* impacting synthesis of *Y*, expression of *Y* in stochastic bursts, and a noisy sensor *Z* that measures *Y* and inhibits it (Fig 2). The overall stochastic system is given by (11), (19) and

$$\begin{array}{cc} & P\left(y\left(t+dt\right)=y\left(t\right)+j|y\left(t\right),x\left(t\right),z\left(t\right)\right)=\frac{k_{y}g\left(z\right)x}{\bar{\left\langle x\right\rangle }}P\left(B_{y}=j\right)dt \end{array}$$ (20a)

$$\begin{array}{cc} & P\left(y\left(t+dt\right)=y\left(t\right)-1|y\left(t\right),x\left(t\right),z\left(t\right)\right)=\gamma_{y}ydt. \end{array}$$ (20b)

#### 2.2.1 Analysis of mean levels

At equilibrium, the mean levels of the random processes *x*(*t*), *y*(*t*) and *z*(*t*) satisfy

$$\begin{array}{cc} & \bar{\left\langle x\right\rangle }=\frac{k_{x}\left\langle B_{x}\right\rangle }{\gamma_{x}},\;\;\bar{\left\langle z\right\rangle }=\frac{k_{z}\left\langle B_{z}\right\rangle \bar{\left\langle y\right\rangle }}{\gamma_{z}},\;\;\frac{k_{y}\langle \bar{g(z)x\rangle }\left\langle B_{y}\right\rangle }{\bar{\left\langle x\right\rangle }}=\gamma_{y}\bar{\left\langle y\right\rangle }. \end{array}$$ (21)

Assuming copy-number fluctuations are tightly regulated by the feedback system, and that they are small,

$$\begin{array}{c} \frac{\bar{\left\langle g(z)x\right\rangle }}{\bar{\left\langle x\right\rangle }}\approx \frac{\bar{\left\langle g(z)\right\rangle }\bar{\left\langle x\right\rangle }}{\bar{\left\langle x\right\rangle }}=\frac{g\left(\bar{\left\langle z\right\rangle }\right)\bar{\left\langle x\right\rangle }}{\bar{\left\langle x\right\rangle }}=g\left(\bar{\left\langle z\right\rangle }\right). \end{array}$$ (22)

Given that *g*(*z*) is a positively-valued monotonically decreasing function, using (21) and (22), the steady-state mean level of *Y* is the unique solution to the equation

$$\begin{array}{c} k_{y}g(\frac{k_{z}\left\langle B_{z}\right\rangle \bar{\left\langle y\right\rangle }}{\gamma_{z}})\left\langle B_{y}\right\rangle =\gamma_{y}\bar{\left\langle y\right\rangle }. \end{array}$$ (23)

Having solved for the means, the burst frequency of *Y* can now be approximated using Taylor series as

$$\begin{array}{c} k_{y}g\left(z\right)x/\bar{\left\langle x\right\rangle }\approx k_{y}g\left(\bar{\left\langle z\right\rangle }\right)(\frac{x}{\bar{\left\langle x\right\rangle }}-f_{p}\frac{z-\bar{\left\langle z\right\rangle }}{\bar{\left\langle z\right\rangle }}) \end{array}$$ (24)

where

$$\begin{array}{c} f_{p}≔-\frac{\bar{\left\langle z\right\rangle }}{g(\bar{\left\langle z\right\rangle })}\frac{dg(z)}{dz}{|}_{z=\bar{\left\langle z\right\rangle }}>0 \end{array}$$ (25)

is the log sensitivity of the function *g* evaluated at steady state. It is important to point out that this linearization of nonlinearities is a key element of the linear noise approximation, and is needed to obtain closed-form solutions to the noise levels [63, 64, 96, 97]. Formulas obtained using this approximation are exact in the limit of small noise, and provide analytical insights into the regulation of noise levels. To see how *f*_*p*_ is regulated by different parameters consider a Hill function for the repression curve

$$\begin{array}{c} g\left(z\right)=\frac{1}{1+{\left(z/z_{c}\right)}^{h}} \end{array}$$ (26)

where *z*_*c*_ denotes the strength of the repression of *Y* by *Z*. In this case the feedback gain is given by

$$\begin{array}{c} f_{p}=h(1-\frac{1}{1+{\left(\bar{\left\langle z\right\rangle }/z_{c}\right)}^{h}}) \end{array}$$ (27)

and is bounded by the Hill coefficient *h*. As can be seen in this equation, *f*_*p*_ can be increased in three different ways: increasing *h*; increasing the mean sensor level $\bar{\langle z\rangle }$; and enhanced repression of *Y* by *Z* (lower *z*_*c*_). Since *k*_*y*_ does not affect *f*_*p*_, it can be modulated to bring the output mean level *Y* to the desired set point.

Note that if the sensor dynamic is very fast compared to the measurand *Y* (i.e., *γ*_*z*_ ≫ *γ*_*y*_), then *z*(*t*) ∝ *y*(*t*), and the burst frequency in (24) will be proportional to the error $y-\bar{\langle y\rangle }$. Hence, this circuit architecture can be interpreted as an approximate proportional controller with feedback gain *f*_*p*_. Finally, if we consider the parameter *k*_*y*_ in *Y*’s burst frequency as an environmental input, then one can define a static sensitivity of $\bar{\langle y\rangle }$ to *k*_*y*_

$$\begin{array}{c} S_{k_{y}}^{\bar{\left\langle y\right\rangle }}≔\frac{k_{y}}{\bar{\left\langle y\right\rangle }}\frac{d\bar{\left\langle y\right\rangle }}{dk_{y}} \end{array}$$ (28)

which using (21) and (25) is given by

$$\begin{array}{c} S_{k_{y}}^{\bar{\left\langle y\right\rangle }}=\frac{1}{1+f_{p}} \end{array}$$ (29)

and monotonically decreases with increasing gain. Note for the open-loop system *f*_*p*_ = 0 and $S_{k_{y}}^{\bar{\langle y\rangle }}=1$ as mean $\bar{\langle y\rangle }$ is simply proportional to *k*_*y*_ from (9).

#### 2.2.2 Analysis of noise levels

Next, we focus on computing the noise levels in *Y* for the overall feedback system. As before, we define a vector *μ* that consists of all the first and second order moments of *x*(*t*), *y*(*t*) and *z*(*t*). The time evolution of *μ* can be obtained by expanding (15) to the three-species system, where *Y*’s nonlinear burst frequency is replaced by its linear approximation (24). Having linear probabilistic rates for all birth-death events results in a linear dynamical system (16) that can be solved analytically to obtain steady-state moments [80]. This analysis yields the following noise level for protein *Y*

$$\begin{array}{cc} CV_{Y}^{2} & =\overset{\text{Intrinsic}\;\text{noise}}{\overset{︷}{\frac{\left(\gamma_{y}+f_{p}\gamma_{y}+\gamma_{z}\right)}{\left(f_{p}+1\right)\left(\gamma_{y}+\gamma_{z}\right)}CV_{int}^{2}}}+\overset{\text{External}\;\text{disturbance}}{\overset{︷}{\frac{\gamma_{y}\left(\left(\gamma_{z}+\gamma_{y}\right)\left(\gamma_{x}+\gamma_{z}\right)+\gamma_{x}\gamma_{y}f_{p}\right)}{\left(1+f_{p}\right)\left(\gamma_{y}+\gamma_{z}\right)\left(\left(\gamma_{x}+\gamma_{y}\right)\left(\gamma_{x}+\gamma_{z}\right)+\gamma_{y}\gamma_{z}f_{p}\right)}CV_{X}^{2}}}\\ & +\overset{\text{Sensor}\;\text{noise}}{\overset{︷}{\frac{f_{p}^{2}\gamma_{y}}{\left(f_{p}+1\right)\left(\gamma_{y}+\gamma_{z}\right)}CV_{Z}^{2}}} \end{array}$$ (30)

which can be decomposed into three components. The first component is the intrinsic noise in *Y* due to its bursty expression, and it decreases with increasing feedback gain *f*_*p*_ approaching a non-zero lower bound $\gamma_{y}CV_{int}^{2}/\left(\gamma_{y}+\gamma_{z}\right)$ as *f*_*p*_ → ∞. This lower bound represents a fundamental limit to which intrinsic noise can be decreased, and this limit is determined by how fast the sensor dynamics is compared to *Y*’s decay rate. The second component is the noise contribution from the external disturbance that monotonically decreases to zero as *f*_*p*_ → ∞. The third component arises from the fact that the sensor *Z* is itself noisy, where

$$\begin{array}{c} CV_{Z}^{2}=\frac{\left\langle B_{z}\right\rangle +\left\langle B_{z}^{2}\right\rangle }{2\left\langle B_{z}\right\rangle \bar{\left\langle z\right\rangle }} \end{array}$$ (31)

is the noise in *Z* due to its own expression occurring in random bursts. This third component is amplified with increasing feedback gain, and as a consequence, the total noise $CV_{Y}^{2}$ is a non-monotonic function of *f*_*p*_ with noise being minimal at an optimal feedback gain (Fig 2). The trade-off between buffering of intrinsic noise and amplification of controller noise is also observed in other biomolecular feedback systems [97]. When *f*_*p*_ = 0, (30) reduces to the open-loop noise (17). Recall that the noise formula (30) is based on the linear noise approximation, and we validate the predicted U-shape noise profile by performing Monte Carlo simulations of the fully stochastic nonlinear system (see S1 Text section I for details).

If the sensor dynamics is sufficiently fast (*γ*_*z*_ ≫ *γ*_*y*_), and the time-scale of disturbance fluctuations are slow (*γ*_*x*_ ≪ *γ*_*y*_), then (30) simplifies to

$$\begin{array}{cc} & CV_{Y}^{2}=\overset{\text{Intrinsic}\;\text{noise}}{\overset{︷}{\frac{1}{1+f_{p}}CV_{int}^{2}}}\;+\overset{\text{External}\;\text{disturbance}}{\overset{︷}{\frac{1}{{\left(1+f_{p}\right)}^{2}}CV_{X}^{2}}}+\overset{\text{Sensor}\;\text{noise}}{\overset{︷}{\frac{f_{p}^{2}\gamma_{y}}{\left(f_{p}+1\right)\gamma_{z}}CV_{Z}^{2}.}} \end{array}$$ (32)

Furthermore, in the absence of the sensor $CV_{Z}^{2}=0$, the resulting noise level (32) corresponds to the scenario where *Y* directly inhibits its expression [38], and in this case $CV_{Y}^{2}$ monotonically decreases with increasing *f*_*p*_. Note that the contribution from external disturbance decreases as $1/f_{p}^{2}$ compared to 1/*f*_*p*_ for the intrinsic noise. Hence, proportional feedback is much more effective in buffering stochasticity from external inputs rather than the intrinsic noise. This point relates to the static sensitivity $S_{k_{y}}^{\bar{\langle y\rangle }}=1/\left(1+f_{p}\right)$ defined in (28), where increasing feedback gain suppresses noise, but it comes at the loss of adapting *Y* levels to changes in the environmental input. Finally, further assuming a strong feedback *f*_*p*_ ≫ 1 in (32), the optimal feedback gain and the corresponding minimal noise level are

$$\begin{array}{cc} & f_{p}={\left(\frac{2CV_{X}^{2}\gamma_{z}}{CV_{Z}^{2}\gamma_{y}}\right)}^{\frac{1}{3}},\;\;CV_{Y}^{2}=3{\left(\frac{CV_{X}CV_{Z}^{2}\gamma_{y}}{2\gamma_{z}}\right)}^{\frac{2}{3}}\;\text{when}\;CV_{int}^{2}=0 \end{array}$$ (33)

$$\begin{array}{cc} & f_{p}=\frac{CV_{int}}{CV_{Z}}\sqrt{\frac{\gamma_{z}}{\gamma_{y}}},\;\;CV_{Y}^{2}=2CV_{int}CV_{Z}\sqrt{\frac{\gamma_{y}}{\gamma_{z}}}\;\text{when}\;CV_{X}^{2}=0, \end{array}$$ (34)

respectively. In summary, while previous works on transcriptional autoregulation have shown monotonically decreasing noise levels with increasing feedback gains [38], implementation of proportional feedback by an intermediate sensor species results in a U-shape noise profile (Fig 2). More specifically, noise from the sensor is amplified at high feedback gains creating a lower limit to which noise can be suppressed as quantified in (33) and (34). Substituting the expressions of *CV*_*int*_ and *CV*_*Z*_ for geometric burst size distributions (see (10)), the lower limit of noise without external disturbance in (34) can be rewritten as

$$\begin{array}{c} CV_{Y}^{2}=\frac{2}{\sqrt{\left(\frac{\bar{\left\langle y\right\rangle }}{\left\langle B_{y}\right\rangle })(\frac{\gamma_{z}\bar{\left\langle z\right\rangle }}{\gamma_{y}\left\langle B_{z}\right\rangle }\right)}}, \end{array}$$ (35)

where $\bar{\langle y\rangle }/\langle B_{y}\rangle$ and $\gamma_{z}\bar{\langle z\rangle }/\left(\gamma_{y}\langle B_{z}\rangle \right)$ represent the average number of burst events within the timescale of $\gamma_{y}^{-1}$ for the target and controller gene respectively. Note that the noise decays as a square root of the number of burst events for controller species *Z* and target species *Y*. This is the same scaling as the fundamental limit of noise suppression in gene expression for any arbitrary controller that was recently quantified [97].

### 2.3 Noise suppression using integral controller

Several biochemical designs of integral feedback controllers have been proposed [98–100], and experimentally implemented in bacterial cells for perfect adaptation in response to environmental perturbations [56]. Naturally existing circuits implementing integral feedback play a key role in regulating cellular heat shock responses [58, 59], and bacterial chemotaxis [60, 61]. We use the stochastic framework to uncover the impact of integral feedback on different noise components of the target protein.

#### 2.3.1 Integral controller design

**Fig 3:**
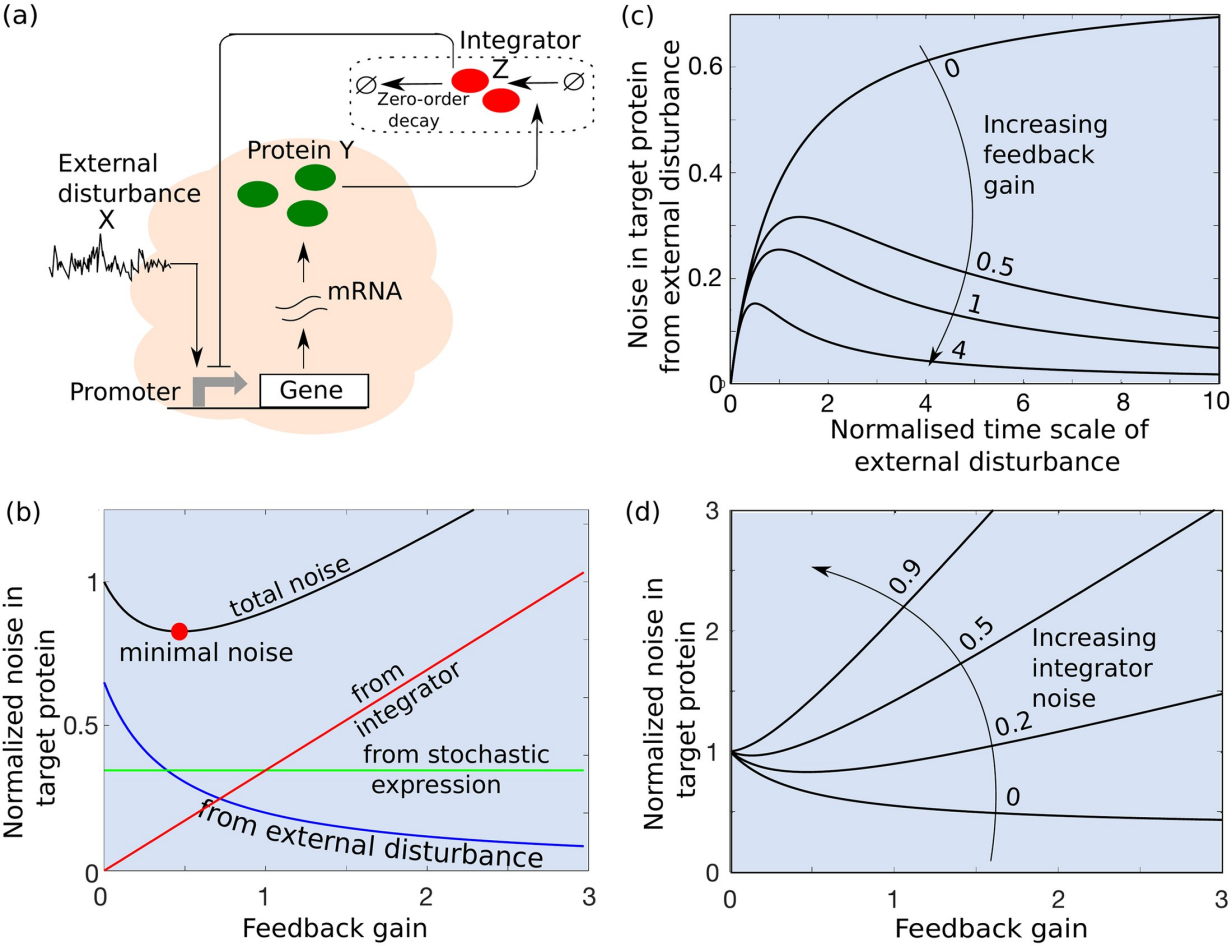
Implementation and noise decomposition for an integral feedback controller. (a) Design of an integral controller where the target protein *Y* activates an integrator species *Z*, *Z* degrades via a zero-order decay process (36) and inhibits the expression of *Y*. (b) Total noise in the target protein (42), and its different noise components as a function of the integral feedback gain. Integral feedback suppresses the noise contribution from external disturbance while keeping the intrinsic noise intact to its open-loop value. The noise contribution from the integrator increases with feedback gain and the total noise is minimized at an intermediate feedback gain. All noise levels are normalized to the total noise at *f*_*i*_ = 0. c) The external noise contribution in (42) varies non-monotonically as a function of the normalized time-scale of disturbance fluctuations *γ*_*y*_/*γ*_*x*_ for different feedback gains. Other parameters taken as $CV_{X}^{2}=0.5$, *k*_*z*_ = *γ*_*y*_, $\bar{\langle z\rangle }/\bar{\langle y\rangle }=1$. (d) The total normalized noise in *Y* with respect to the feedback gain for different integrator noise $CV_{Z}^{2}$. For this plot parameters taken as $CV_{X}^{2}=0.5$, *γ*_*y*_ = 3*γ*_*x*_ and $CV_{int}^{2}=0.2$.

We consider the simplest controller design where an integrator species *Z* is activated by the target protein *Y*, and degrades via a zero-order decay process (Fig 3). More specifically, in the deterministic limit the dynamics of *z* is given by

$$\begin{array}{c} \frac{dz(t)}{dt}=k_{z}\left(y\left(t\right)-\bar{\left\langle y\right\rangle }\right), \end{array}$$ (36)

where the *net* decay of *Z* per unit time is a constant and equal to $k_{z}\bar{\langle y\rangle }$, leading to an integration of the error between *y*(*t*) and its given target set point $\bar{\langle y\rangle }$. The realization of such a scheme follows straightforwardly from Michaelis-Menten enzyme kinetics. Inside cells proteins are actively degraded by enzymes known as proteases. When the enzyme is present in high abundance compared to the substrate (protein *Z*), then decay is a first-order process that is proportional to the protein level *z*(*t*). In contrast, when the substrate is present in sufficiently high abundance, then decay follows a zero-order process that becomes invariant of the protein level, and is determined by the enzyme level. In essence, the rate-limiting enzyme sets the constant decay in (36), which in turn determines the target protein’s set point.

An alternative implementation is proposed in [98] with deterministic dynamics

$$\begin{array}{c} \frac{dz(t)}{dt}=k_{z}z\left(t\right)(\bar{\left\langle y\right\rangle }-y\left(t\right)) \end{array}$$ (37)

that is realized by *Z* activating its own expression, and *Y* degrading *Z* via a first-order process. It turns out that both these implementations lead to similar noise levels in the target protein, and we focus our efforts on the simpler implementation through a zero-order decay.

#### 2.3.2 Analysis of noise levels

In the stochastic setting of (36), the time evolution of integer-valued *z*(*t*) is modeled as a bursty birth-death process

$$\begin{array}{cc} & P\left(z\left(t+dt\right)=z+j|z\left(t\right)=z\right)=\frac{k_{z}y}{\left\langle B_{z}\right\rangle }P\left(B_{z}=j\right)dt, \end{array}$$ (38a)

$$\begin{array}{cc} & P\left(z\left(t+dt\right)=z-1|z\left(t\right)=z\right)=k_{z}\bar{\left\langle y\right\rangle }dt \end{array}$$ (38b)

with the degradation-event probability now being a constant and proportional to the target protein’s desired set point. As before, we redefine the burst frequency of *Y* to be $k_{y}g\left(z\right)x\left(t\right)/\bar{\langle x\rangle }$ to include the repression by *Z*, where *g*(*z*) is a positively-valued monotonically decreasing function of *z*(*t*). The stochastic dynamics of *y*(*t*) and the external disturbance *x*(*t*) are given by (20) and (11), respectively. Exploiting the linear noise approximation, we linearize *Y*’s burst frequency

$$\begin{array}{c} k_{y}g\left(z\right)x/\bar{\left\langle x\right\rangle }\approx k_{y}g\left(\bar{\left\langle z\right\rangle }\right)(\frac{x}{\bar{\left\langle x\right\rangle }}-f_{i}\frac{z-\bar{\left\langle z\right\rangle }}{\bar{\left\langle z\right\rangle }}) \end{array}$$ (39)

$$\begin{array}{c} f_{i}≔-\frac{\bar{\left\langle z\right\rangle }}{g(\bar{\left\langle z\right\rangle })}\frac{dg(z)}{dz}{|}_{z=\bar{\left\langle z\right\rangle }}>0 \end{array}$$ (40)

where *f*_*i*_ is the integral feedback gain, and $\bar{\langle z\rangle }$ is the unique solution to

$$\begin{array}{c} k_{y}g(\bar{\left\langle z\right\rangle })\left\langle B_{y}\right\rangle =\gamma_{y}\bar{\left\langle y\right\rangle }. \end{array}$$ (41)

Note by integral feedback design the mean levels of *y*(*t*) converge to $\bar{\langle y\rangle }$ over time, hence the static sensitivity of $\bar{\langle y\rangle }$ to *k*_*y*_ is $S_{k_{y}}^{\bar{\langle y\rangle }}=0$.

Performing a stochastic analysis as described in the previous section yields the following steady-state noise level in the target protein

$$\begin{array}{cc} CV_{Y}^{2}= & \overset{\text{External}\;\text{disturbance}}{\overset{︷}{\frac{\bar{\left\langle z\right\rangle }\gamma_{x}\gamma_{y}}{\left(\bar{\left\langle y\right\rangle }f_{i}k_{z}\gamma_{y}+\bar{\left\langle z\right\rangle }\left(\gamma_{y}\gamma_{x}+\gamma_{x}^{2}\right)\right)}CV_{X}^{2}}}+\overset{\text{Integrator}}{\overset{︷}{f_{i}CV_{Z}^{2}}}+\overset{\text{Intrinsic}\;\text{noise}}{\overset{︷}{CV_{int}^{2}}} \end{array}$$ (42)

where $CV_{int}^{2}$ and $CV_{Z}^{2}$ are given by (17) and (31), respectively.

The first noise component in (42) represents the noise contribution from the external disturbance, and interestingly, it varies non-monotonically with the time-scale of disturbance *γ*_*x*_ (Fig 3). With increasing *γ*_*x*_, $CV_{Y}^{2}$ first increases to reach a maximum when

$$\begin{array}{c} \gamma_{x}=\sqrt{\frac{k_{z}f_{i}\bar{\left\langle y\right\rangle }\gamma_{y}}{\bar{\left\langle z\right\rangle }}}, \end{array}$$ (43)

and then decreases to zero as *γ*_*x*_ is increased beyond (43). This behavior can be contrasted to the open-loop system (17) where this noise component monotonically decreases to zero with increasing *γ*_*x*_. Intuitively, the non-monotonicity arises because the integral feedback is able to compensate slowly-varying disturbances allowing adaptation of *y*(*t*) to $\bar{\langle y\rangle }$, while rapidly-varying disturbances are effectively buffered by time averaging as in the open-loop system. The second noise component in (42) represents the contribution from *Z*’s stochastic expression, and it increases with stronger feedbacks due to enhanced noise propagation from *Z* to *Y*. Finally, the intrinsic noise in the target protein remains unaltered by integral feedback. For slowly-varying disturbances (*γ*_*x*_ → 0), (42) reduces to

$$\begin{array}{cc} CV_{Y}^{2}= & \overset{\text{External}\;\text{disturbance}}{\overset{︷}{\frac{\bar{\left\langle z\right\rangle }\gamma_{x}}{\bar{\left\langle y\right\rangle }f_{i}k_{z}}CV_{X}^{2}}}+\overset{\text{Integrator}}{\overset{︷}{f_{i}CV_{Z}^{2}}}+\overset{\text{Intrinsic}\;\text{noise}}{\overset{︷}{CV_{int}^{2}}} \end{array}$$ (44)

illustrating the differential scaling of noise components with *f*_*i*_ (Fig 3). In this limit, the minimal noise level

$$\begin{array}{c} CV_{Y}^{2}=2\sqrt{\frac{\bar{\left\langle z\right\rangle }\gamma_{x}}{\bar{\left\langle y\right\rangle }k_{z}}}CV_{X}CV_{Z}+CV_{int}^{2} \end{array}$$ (45)

is achieved at an optimal feedback gain

$$\begin{array}{c} f_{i}=\sqrt{\frac{\bar{\left\langle z\right\rangle }\gamma_{x}}{\bar{\left\langle y\right\rangle }k_{z}}}\frac{CV_{X}}{CV_{Z}}. \end{array}$$ (46)

Note that the minimal noise scales as $CV_{Y}^{2}\propto CV_{X}$ for integral feedback, but scales as $CV_{Y}^{2}\propto CV_{X}^{\frac{2}{3}}$ for proportional feedback in (33). Thus, while the proportional feedback may provide better scaling of $CV_{Y}^{2}$ with *CV*_*X*_, the scaling factor for integral feedback can be arbitrarily reduced by increasing *k*_*z*_.

The alternative implementation of integral feedback (37) leads to similar noise level

$$\begin{array}{cc} CV_{Y}^{2}= & \overset{\text{External}\;\text{disturbance}}{\overset{︷}{\frac{\gamma_{x}\gamma_{y}}{\left(\bar{\left\langle y\right\rangle }f_{i}k_{z}\gamma_{y}+\gamma_{y}\gamma_{x}+\gamma_{x}^{2}\right)}CV_{X}^{2}}}+\overset{\text{Integrator}}{\overset{︷}{f_{i}CV_{Z}^{2}}}+\overset{\text{Intrinsic}\;\text{noise}}{\overset{︷}{CV_{int}^{2}}} \end{array}$$ (47)

with the only difference being in some proportionality constants to match dimensionality in the first noise component. In summary, the results show that an integral controller selectively buffers the noise from external disturbance while amplifying the noise from the controller, and the noise buffering is most effective for a slowly-varying external disturbance [60]. Finally, we point out that the predicted U-shape noise profile in Fig 3B is confirmed using stochastic simulations of the nonlinear integral feedback design (see S1 Text section I for details).

### 2.4 Noise suppression using derivative controller

Having completed the analysis for proportional and integral controllers we next turn our attention to a derivative controller.

#### 2.4.1 Derivative controller design

How can biochemical circuits be designed to approximately sense the derivative of *y*(*t*)? To see this, consider the sensor dynamics in the deterministic limit

$$\begin{array}{c} \frac{dz(t)}{dt}=k_{z}\left\langle B_{z}\right\rangle y\left(t\right)-\gamma_{z}z\left(t\right) \end{array}$$ (48)

which in the Laplace domain corresponds to

$$\begin{array}{c} Z\left(s\right)=\frac{k_{z}\left\langle B_{z}\right\rangle Y\left(s\right)}{s+\gamma_{z}}=\frac{\bar{\left\langle z\right\rangle }}{\bar{\left\langle y\right\rangle }}\frac{Y(s)}{\frac{s}{\gamma_{z}}+1}. \end{array}$$ (49)

**Fig 4:**
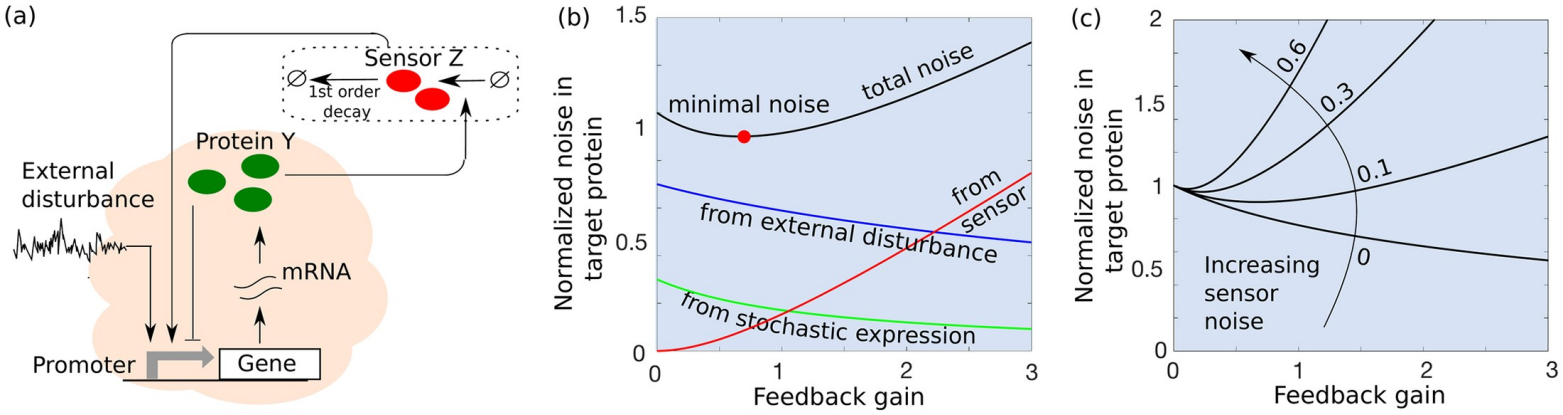
Implementation and noise decomposition for a derivative-based controller. (a) Schematic of the derivative controller where *Y* activates the sensor *Z*, *Z* activates the burst frequency of *Y*, while *Y* represses its own burst frequency (b) Different noise components in (57) are plotted as a function of the derivative feedback gain *f*_*d*_. Noise levels are normalized by the open-loop noise (17) and other parameters are chosen as $CV_{int}^{2}=0.25$, $CV_{Z}^{2}=0.1$, $CV_{X}^{2}=0.7$, $\gamma_{x}=\frac{1}{5}\gamma_{y}$, *γ*_*z*_ = 3*γ*_*y*_. While both the intrinsic noise, and the noise contribution from the external disturbance decrease with increasing *f*_*d*_, the noise contribution from the sensor increases. In contrast to the proportional feedback, the intrinsic noise decreases faster than the disturbance contribution. (c) The noise in *Y* as a function of the derivative feedback gain *f*_*d*_ emphasizes the nonmonotonic noise profile for different levels of sensor noise $CV_{Z}^{2}$.

Here *Z*(*s*) and *Y*(*s*) are the Laplace transforms of *z*(*t*) and *y*(*t*), respectively, and we have used the fact that at equilibrium $\bar{\langle z\rangle }/\bar{\langle y\rangle }=k_{z}\langle B_{z}\rangle /\gamma_{z}$. The burst frequency function *g*(*y*, *z*) must obey particular parametric constraints to act as a derivative controller (see S1 Text section III for details). In this manuscript, we consider the scenario where *Y* inhibits its own burst frequency and *Z* activates it (Fig 4). A general form for *g*(*y*, *z*) is given by the product of activation and repression Hill functions [95] as

$$\begin{array}{c} g\left(y,z\right)=(\frac{{\left(z/z_{c}\right)}^{h_{z}}}{1+{\left(z/z_{c}\right)}^{h_{z}}})(\frac{1}{1+{\left(y/y_{c}\right)}^{h_{y}}}). \end{array}$$ (50)

Here, *y*_*c*_ and *z*_*c*_ are the levels of *Y* and *Z* for the half-maximal repression and half-maximal activation, while the Hill coefficients are *h*_*y*_ and *h*_*z*_ respectively. For strong binding affinity of *Y* and weak affinity of *Z* represented by $y_{c}\ll \bar{\langle y\rangle }$ and $z_{c}\gg \bar{\langle z\rangle }$, we get the relationship that the Hill coefficients must be same *h*_*z*_ = *h*_*y*_ = *h* for derivative control [95, 101]. The subsequent form of the burst frequency function being proportional to (*z*/*y*)^*h*^ leads to a simpler analysis in terms of number of parameters. An arbitrary choice of *h*_*y*_, *h*_*z*_, *y*_*c*_, and *z*_*c*_ can lead to a combination of derivative and proportional controllers (see S1 Text section III for details) Then, in the limit of small fluctuations in *z*(*t*), *y*(*t*) around $\bar{\langle z\rangle }$, $\bar{\langle y\rangle }$, respectively,

$$\begin{array}{c} {\left(\frac{z}{y}\right)}^{h}-{\left(\frac{\bar{\left\langle z\right\rangle }}{\bar{\left\langle y\right\rangle }}\right)}^{h}\approx h{\left(\frac{\bar{\left\langle z\right\rangle }}{\bar{\left\langle y\right\rangle }}\right)}^{h}(\frac{z}{\bar{\left\langle z\right\rangle }}-\frac{y}{\bar{\left\langle y\right\rangle }}). \end{array}$$ (51)

Using (49) and assuming *γ*_*z*_ is large (i.e., fast sensor dynamics), the Laplace transform of the right-hand-side of (51) is

$$\begin{array}{cc} & h{\left(\frac{\bar{\left\langle z\right\rangle }}{\bar{\left\langle y\right\rangle }}\right)}^{h}(\frac{Z(s)}{\bar{\left\langle z\right\rangle }}-\frac{Y(s)}{\bar{\left\langle y\right\rangle }})=-{\left(\frac{\bar{\left\langle z\right\rangle }}{\bar{\left\langle y\right\rangle }}\right)}^{h}(\frac{hsY(s)}{\bar{\left\langle y\right\rangle }\left(s+\gamma_{z}\right)}) \end{array}$$ (52a)

$$\begin{array}{c} \approx -{\left(\frac{\bar{\left\langle z\right\rangle }}{\bar{\left\langle y\right\rangle }}\right)}^{h}(\frac{hsY(s)}{\bar{\left\langle y\right\rangle }\gamma_{z}}). \end{array}$$ (52b)

Recall that *sY*(*s*) is the Laplace transform of the time derivate of *y*(*t*), and hence in the time-domain, the burst frequency (51) corresponds to implementing a derivative controller. Going back to the original stochastic system, let the frequency of bursts in the *Y* protein be given by $k_{y}x/\bar{\langle x\rangle }{\left(z/y\right)}^{h}$. Then, as the ratio $\bar{\langle z\rangle }/\bar{\langle y\rangle }=k_{z}\langle B_{z}\rangle /\gamma_{z}$ becomes a constant at steady-state, the mean protein level for *Y*

$$\begin{array}{c} \bar{\left\langle y\right\rangle }=\frac{k_{y}\left\langle B_{y}\right\rangle }{\gamma_{y}}\bar{{\left(\frac{z}{y}\right)}^{h}}\approx \frac{k_{y}\left\langle B_{y}\right\rangle }{\gamma_{y}}{\left(\frac{\bar{\left\langle z\right\rangle }}{\bar{\left\langle y\right\rangle }}\right)}^{h}=\frac{k_{y}\left\langle B_{y}\right\rangle }{\gamma_{y}}{\left(\frac{k_{z}\left\langle B_{z}\right\rangle }{\gamma_{z}}\right)}^{h} \end{array}$$ (53)

is proportional to *k*_*y*_ and the sensitivity $S_{k_{y}}^{\bar{\langle y\rangle }}=1$ as in the open-loop system. Note that unlike the proportional controller design in Fig 2, here we have *Z* activating *Y*’s expression, while *Y* inhibits its own expression. This dual-control of expression using both species is a critical feature of the derivative controller that allows an estimate of the rate of change of the output by performing a molecular subtraction of the current output (*Y*) with the delayed output (*Z*).

#### 2.4.2 Analysis of noise levels

To perform a noise analysis of the derivative controller-based feedback system, we revert to the noisy sensor *Z* described by the bursty birth-death process (19). The stochastic dynamics of protein *Y* is now described by

$$\begin{array}{c} P(y(t+dt)=y\left(t\right)+j|y\left(t\right),x\left(t\right),z\left(t\right))=k_{y}\frac{x}{\bar{\left\langle x\right\rangle }}{\left(\frac{z}{y}\right)}^{h}P\left(B_{y}=j\right)dt \end{array}$$ (54a)

$$\begin{array}{c} P(y(t+dt)=y\left(t\right)-1|y\left(t\right),x\left(t\right),z\left(t\right))=\gamma_{y}ydt. \end{array}$$ (54b)

As before, the external disturbance is described by (11). To write a closed systems of differential equations for the time evolution of moments, we linearize protein *Y*’s burst frequency assuming small copy-number fluctuations

$$\begin{array}{c} k_{y}\frac{x}{\bar{\left\langle x\right\rangle }}{\left(\frac{z}{y}\right)}^{h}\approx k_{y}{\left(\frac{\bar{\left\langle z\right\rangle }}{\bar{\left\langle y\right\rangle }}\right)}^{h}(\frac{x}{\bar{\left\langle x\right\rangle }}+\frac{f_{d}\gamma_{z}}{\gamma_{y}}(\frac{z}{\bar{\left\langle z\right\rangle }}-\frac{y}{\bar{\left\langle y\right\rangle }})) \end{array}$$ (55)

where

$$\begin{array}{c} f_{d}≔\frac{h\gamma_{y}}{\gamma_{z}}>0. \end{array}$$ (56)

is the derivative feedback gain. A steady-state analysis of the resulting linear moment dynamics yields the following noise in protein *Y*

$$\begin{array}{cc} CV_{Y}^{2} & =\overset{\text{Intrinsic}\;\text{noise}}{\overset{︷}{\frac{\left(\gamma_{y}+\gamma_{z}\right)}{\gamma_{y}+\gamma_{z}f_{d}+\gamma_{z}}CV_{int}^{2}}}+\overset{\text{External}\;\text{disturbance}}{\overset{︷}{\frac{\gamma_{y}\left(\gamma_{y}\left(\gamma_{x}+\gamma_{z}\right)+\gamma_{z}\left(\gamma_{x}+\gamma_{z}+\gamma_{z}f_{d}\right)\right)}{\left(\gamma_{y}+\gamma_{z}f_{d}+\gamma_{z}\right)\left(\gamma_{y}\left(\gamma_{x}+\gamma_{z}\right)+\gamma_{x}\left(\gamma_{x}+\gamma_{z}f_{d}+\gamma_{z}\right)\right)}CV_{X}^{2}}}\\ & +\overset{\text{Sensor}\;\text{noise}}{\overset{︷}{\frac{f_{d}^{2}\gamma_{z}^{2}}{\gamma_{y}\left(\gamma_{y}+\gamma_{z}f_{d}+\gamma_{z}\right)}CV_{Z}^{2}.}} \end{array}$$ (57)

**Fig 5:**
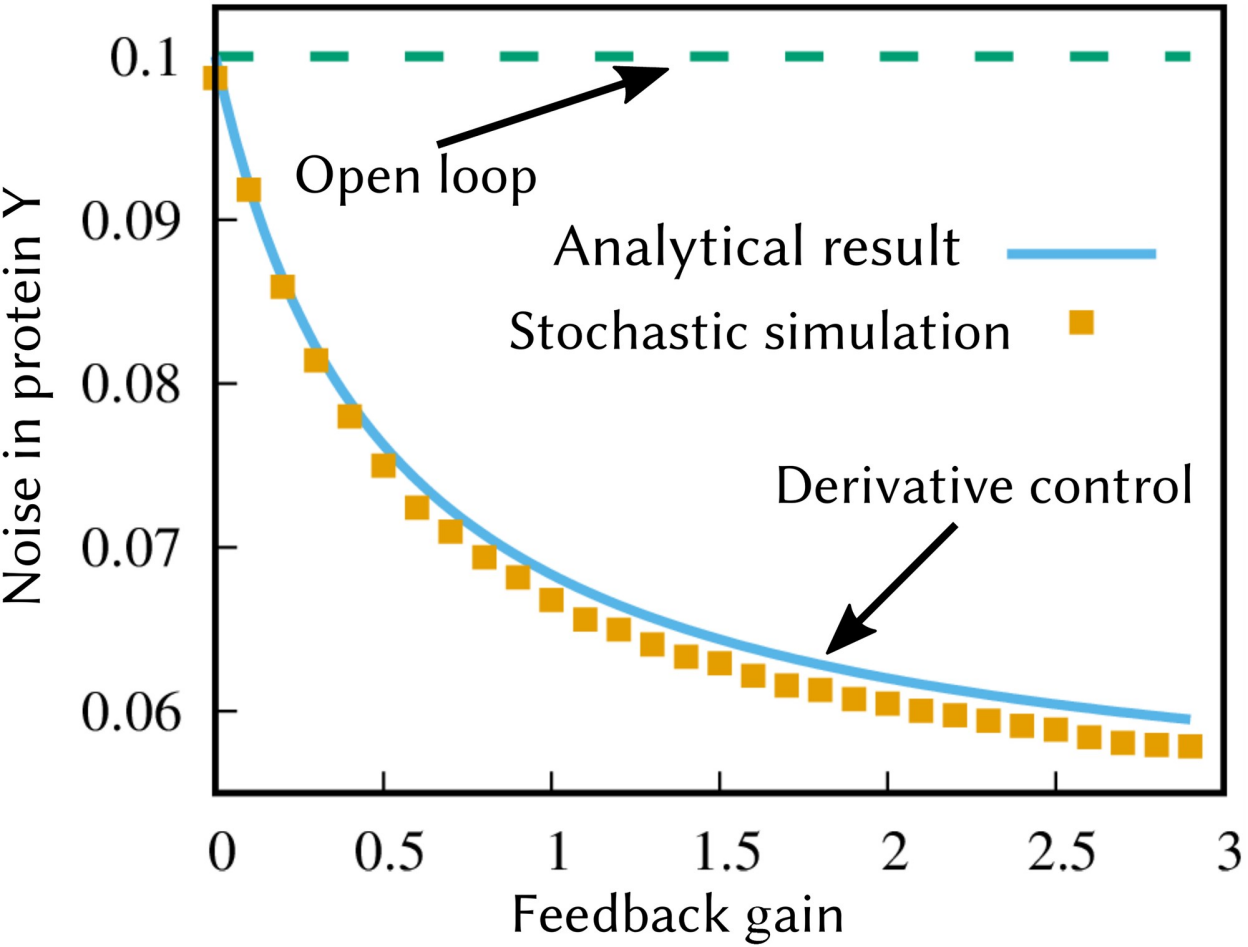
A derivative-based controller minimizes stochastic fluctuations in protein levels. Noise in the level of protein *Y* as obtained by performing exact Monte Carlo simulations of the nonlinear feedback system (19) and (54) with *h* = 1 and no external disturbance $x=\bar{\langle x\rangle }$ with probability one. Other parameters are taken as *k*_*y*_ = 2, *γ*_*y*_ = 0.2, 〈*B*_*y*_〉 = 20, 〈*B*_*z*_〉 = 1, *k*_*z*_ = *γ*_*z*_, with burst sizes following a shifted geometric distribution (see S1 Text section I). In this case, *γ*_*z*_ was varied to change the gain *f*_*d*_ as per (56). The noise level obtained by running a large number of Monte Carlo simulations match their analytical estimates in (57).

Analysis of the resulting noise components reveals that both the intrinsic noise, and the noise contribution from the external disturbance, decrease with increasing gain *f*_*d*_, with the former showing a much faster decay (Fig 4). The noise contribution from the sensor amplifies with increasing feedback gain resulting in the total noise $CV_{Y}^{2}$ being minimized at an intermediate gain (Fig 4). In comparison to the proportional and integral controllers, if adaptation of *Y* level to the environmental input is desired, then the derivative controller is optimal as it offers qualitatively similar noise buffering without affecting static sensitivity. This is because in the proportional controller, sensitivity reduces with increasing feedback strength as in (29) and in the integral controller, the sensitivity is zero. In the limit of slow fluctuations in the external disturbance *γ*_*x*_ ≪ *γ*_*y*_, *γ*_*z*_, the above noise level simplifies to

$$\begin{array}{cc} CV_{Y}^{2} & =\overset{\text{Intrinsic}\;\text{noise}}{\overset{︷}{\frac{\left(\gamma_{y}+\gamma_{z}\right)}{\gamma_{y}+\gamma_{z}f_{d}+\gamma_{z}}CV_{int}^{2}}}\;+\overset{\text{External}\;\text{disturbance}}{\overset{︷}{CV_{X}^{2}}}+\overset{\text{Sensor}\;\text{noise}}{\overset{︷}{\frac{f_{d}^{2}\gamma_{z}^{2}}{\gamma_{y}\left(\gamma_{y}+\gamma_{z}f_{d}+\gamma_{z}\right)}CV_{Z}^{2}}} \end{array}$$ (58)

showing the derivative controller’s inability to reject low-frequency external disturbances. Finally, assuming no external disturbance ($CV_{X}^{2}=0$), we verify the ability of a derivative controller to minimize intrinsic noise in *Y* by performing exact Monte Carlo simulations based on the Stochastic Simulation Algorithm (SSA) [102]. Stochastic simulation results of the overall nonlinear feedback system are shown in Fig 5, and the noise levels show a good match with the formula (57) confirming the noise suppression abilities of a derivative controller.

## 3 Discussion

While PID controllers have become quite standard in industry, designing biochemical circuits that perform analogous functions inside cells is a highly nontrivial problem. Generally, controllers operate with all PID components as they are designed considering multiple constraints (system stability, transient response, disturbance rejection, error between measured and desired output). However, in this contribution we have focused on a single output—minimizing deleterious fluctuations in the protein level around a desired set point. To obtain insights into this process, we investigate controllers individually and gauge their performance with respect to noise mitigating.

Here we present simple circuits that function as *approximate* PID controllers assuming fluctuations in molecular counts are small around their respective means. Analytical formulas for protein noise levels were obtained using the linear noise approximation, and while these formulas are exact in the small-noise regime, they may provide poor quantitative fits in parameter regimes leading to large fluctuations in species copy numbers (see S1 Text section I and II for details).

The realization of an approximate proportional controller can arise in naturally occurring autorepressive motifs which are abundant in *E. coli* [48, 103]. In this case, the feedback system has two species, mRNA and the protein translated from it. The protein then acts like the sensor species (*Z*) actuating proportional control of mRNA levels with mRNA being the target species (*Y*). Our analysis of biochemically-implemented proportional feedback reveals the following properties:

- Proportional feedback is more efficient in suppressing stochasticity arising from noisy input signals, compared to noise arising from protein expression occurring in random bursts (Fig 2).
- Any form of measurement noise (for example, due to stochastic expression of the sensor protein), leads to an optimal feedback gain for minimizing total protein noise via a U-shape profile. A similar U-shaped curve for noise in gene expression is observed in experiments with increasing feedback strength, which is reminiscent of the trade-off between buffering of intrinsic noise and amplification of sensor noise (Fig 2B) [37].
- Consistent with prior work [104], noise suppression in the proportional feedback circuit comes at the cost of reduced static input-output sensitivity, i.e., the protein levels are precisely regulated for a given environment, but do not respond to new environments.
- For our specific proportional controller, the minimum target protein noise obeys the fundamental limit of noise suppression obtained in [97] for an arbitrary controller (see (35)).

Integral controllers are commonly found in natural regulatory circuits and known for their robust adaptive response [58, 61]. They also have been recently implemented in synthetic circuits [56, 57]. However, the stochastic behaviour of the integral controller is not clear. Here, we have shown that, in contrast to proportional feedback controllers, integral controllers are highly efficient in suppressing slow external disturbances, and have no impact on the noise contribution from *Y*’s bursty expression (Fig 3). Interestingly, the noise contribution from external disturbances was found to be maximized at intermediate frequencies of the disturbance. While the above analysis was restricted to single controllers, it begs the question if a combination of different controllers can provide better noise suppression. Our analysis of a PI (mixture of proportional and integral) controller shows that in the presence of extrinsic noise a PI controller provides better noise suppression compared to individual controllers (see S1 Text section II for details).

The derivative controllers are not well known in biology. Biochemical designs of derivative control have been proposed in theoretical works [62]. The purpose of derivative controller is to buffer fast changing disturbances by estimating of the rate of change of the output. In our work, this rate of change is estimated by performing a molecular subtraction of the current output (*Y*) with the delayed output (*Z*), which is an important feature of incoherent feedforward loops. Incoherent feedforward loops have two arms, one repressing and one activating a target species. One of the arms contains an intermediate species while the other is a direct regulation of the target species. The existence of the intermediate species creates a delay in target species regulation with respect to the direct regulation creating the aforementioned molecular subtraction. Typically in the feedforward configuration, the source of the regulation is different from the target species. However, in this work we consider the simplified case where incoherent regulation is fed back on to *Y* itself to give derivative action. Incoherent feedforward motifs are known to be abundant in *E. coli* and higher organisms [105–108]. One can speculate that at least some of these motifs are part of feedback circuits, however a rigorous bioinformatic analysis would have to be done to show this. Intriguingly, our analysis shows that this controller suppresses intrinsic noise in the protein while preserving the open-loop static input-output sensitivity (see (Eq 53)). Intuitively, any rapid increase in protein levels due to a random burst is compensated by lowering the frequency of subsequent bursts. We confirmed our small-noise analysis results with exact Monte Carlo simulations of the nonlinear feedback system (Fig 5).

The feedback gain in the controllers depends mainly on the two circuit elements: the Hill coefficient *h*, denoting the sensitivity of the regulation of *Y* by *Z* and the mean level of the sensor species $\bar{\langle z\rangle }$, reflecting the strength of *Y* → *Z* activation. In the above noise plots, we, however, have kept $\bar{\langle z\rangle }$ constant and have mainly varied *h*. Alternatively, one could also change $\bar{\langle z\rangle }$ to vary the feedback gain. A natural question arises: Do the noise properties of *Y* depend on the way feedback gain is varied? To understand the effect of *Y* → *Z* activation strength, we plot the target protein noise as a function of $\bar{\langle z\rangle }$ for all the controllers (see S1 Text section IV for details). We observe that the proportional controller mostly acts as a noise buffer as a function of $\bar{\langle z\rangle }$, except for a large *h* value where increasing $\bar{\langle z\rangle }$ enhances the sensor noise contribution. In the case of integral controller, increasing *Y* to *Z* activation strength is not that effective to reduce the noise in the target protein as the increment of $\bar{\langle z\rangle }$ can lead to enhancement of both the sensor and extrinsic noise components. For the derivative controller, the target noise reduces asymptotically with $\bar{\langle z\rangle }$. However, the controller is ineffective in buffering intrinsic noise component by increasing $\bar{\langle z\rangle }$ compared to increasing *h* as this noise remains unchanged. Thus, the increment of activation strength of *Y* to *Z* or the repression Hill coefficient can reduce the total target protein noise in a range of parameter values and their noise reduction ability depends on the context.

As part of future work, we will investigate design of controller that consider a combination of proportional, integral and derivative controllers for regulating multiple system properties at the same time, for example maintaining a given static input-output sensitivity, noise in the target protein, transient response, and noise propagation to downstream proteins. Our study of the derivative controller has a limitation as the Hill coefficient is the only tunable parameter for feedback gain. This is because of our specific choice of the burst frequency function of *Y* has the same Hill coefficients for both activation and repression in the weak binding limit of *Z* and strong binding limit of *Y* to the *Y* promoter. While this choice gives us theoretical insights through simple analytical expressions of noise, the analysis with multiple tunable parameters (as in [62, 109]) is a matter of our future study. Another direction is to study information processing in biochemical controllers using the framework of channel capacity, i.e., how many input states can be accurately discriminated form a noisy output of the target protein. Our preliminary work shows that proportional controllers decreases channel capacity in spite of noise suppression due to the reduced input-output sensitivity [110].

## Supporting information

S1 Text Supplementary text includes I) Comparison of analytical results with stochastic simulations; II) Stochastic simulation for a P and I combination circuit; III) Parametric constraints for derivative control; IV) Noise plots with changing feedback gain via *Y* to *Z* activation strength; V) Considering external disturbance as an OU process. In addition, Fig A shows the analytical vs simulation results for the proportional controller, Fig B shows the analytical vs simulation results for integral controller, and Fig C shows the simulation results for the combined circuit of proportional and integral controller. Fig D shows the effect of changing the activation of *Z* by *Y* on total noise.
(PDF)

## References

[1] Raser JM, O’Shea EK. Noise in Gene Expression: Origins, Consequences, and Control. Science. 2005;309:2010—2013. doi: 10.1126/science.1105891 16179466 PMC1360161

[2] Elowitz MB, Levine AJ, Siggia ED, Swain PS. Stochastic Gene Expression in a Single Cell. Science. 2002;297:1183–1186. doi: 10.1126/science.1070919 12183631

[3] Bar-Even A, Paulsson J, Maheshri N, Carmi M, O’Shea E, Pilpel Y, et al. Noise in protein expression scales with natural protein abundance. Nature Genetics. 2006;38:636–643. doi: 10.1038/ng1807 16715097

[4] Blake WJ, Kaern M, Cantor CR, Collins JJ. Noise in eukaryotic gene expression. Nature. 2003;422:633–637. doi: 10.1038/nature01546 12687005

[5] Blake WJ, Balazsi G, Kohanski MA, Isaacs FJ, Murphy KF, Kuang Y, et al. Phenotypic Consequences of Promoter-Mediated Transcriptional Noise. Molecular Cell. 2006;24:853–865. doi: 10.1016/j.molcel.2006.11.003 17189188

[6] Newman JRS, Ghaemmaghami S, Ihmels J, Breslow DK, Noble M, DeRisi JL, et al. Single-cell proteomic analysis of S. cerevisiae reveals the architecture of biological noise. Nature Genetics. 2006;441:840–846. 16699522 10.1038/nature04785

[7] Libby E, Perkins TJ, Swain PS. Noisy information processing through transcriptional regulation. Proceedings of the National Academy of Sciences. 2007;104:7151–7156. doi: 10.1073/pnas.0608963104 17420464 PMC1855426

[8] Cook DL, Gerber AN, Tapscott SJ. Modeling stochastic gene expression: implications for haploinsufficiency. Proceedings of the National Academy of Sciences. 1998;95:15641–15646. doi: 10.1073/pnas.95.26.15641 9861023 PMC28097

[9] Kemkemer R, Schrank S, Vogel W, Gruler H, Kaufmann D. Increased noise as an effect of haploinsufficiency of the tumor-suppressor gene neurofibromatosis type 1 in vitro. Proceedings of the National Academy of Sciences. 2002;99:13783–13788. doi: 10.1073/pnas.212386999 12368469 PMC129775

[10] Eldar A, Elowitz MB. Functional roles for noise in genetic circuits. Nature. 2010;467:167–173. doi: 10.1038/nature09326 20829787 PMC4100692

[11] Wang T, Dunlop MJ. Controlling and exploiting cell-to-cell variation in metabolic engineering. Current Opinion in Biotechnology. 2019;57:10–16. doi: 10.1016/j.copbio.2018.08.013 30261323

[12] Roeder AHK. Use it or average it: stochasticity in plant development. Current Opinion in Plant Biology. 2018;41:8–15. doi: 10.1016/j.pbi.2017.07.010 28837855

[13] Singh A, Weinberger LS. Stochastic gene expression as a molecular switch for viral latency. Current Opinion in Microbiology. 2009;12:460–466. doi: 10.1016/j.mib.2009.06.016 19595626 PMC2760832

[14] Norman TM, Lord ND, Paulsson J, Losick R. Memory and modularity in cell-fate decision making. Nature. 2013;503:481–486. doi: 10.1038/nature12804 24256735 PMC4019345

[15] Maamar H, Raj A, Dubnau D. Noise in Gene Expression Determines Cell Fate in Bacillus subtilis. Science. 2007;317:526–529. doi: 10.1126/science.1140818 17569828 PMC3828679

[16] Balázsi G, van Oudenaarden A, Collins JJ. Cellular Decision Making and Biological Noise: From Microbes to Mammals. Cell. 2014;144:910–925. 10.1016/j.cell.2011.01.030 PMC3068611 21414483

[17] Chang HH, Hemberg M, Barahona M, Ingber DE, Huang S. Transcriptome-wide noise controls lineage choice in mammalian progenitor cells. Nature. 2008;453:544–547. doi: 10.1038/nature06965 18497826 PMC5546414

[18] Veening JW, Smits WK, Kuipers OP. Bistability, Epigenetics, and Bet-Hedging in Bacteria. Annual Review of Microbiology. 2008;62:193–210. doi: 10.1146/annurev.micro.62.081307.163002 18537474

[19] Norman TM, Lord ND, Paulsson J, Losick R. Stochastic Switching of Cell Fate in Microbes. Annual Review of Microbiology. 2015;69:381–403. doi: 10.1146/annurev-micro-091213-112852 26332088

[20] Vasdekis AE, Alanazi H, Silverman AM, Williams C, Canul AJ, Cliff JB, et al. Eliciting the impacts of cellular noise on metabolic trade-offs by quantitative mass imaging. Nature communications. 2019;10:848. doi: 10.1038/s41467-019-08717-w 30783105 PMC6381102

[21] Lycus P, Soriano-Laguna MJ, Kjos M, Richardson DJ, Gates AJ, Milligan DA, et al. A bet-hedging strategy for denitrifying bacteria curtails their release of N2O. Proceedings of the National Academy of Sciences. 2018;115(46):11820–11825. doi: 10.1073/pnas.1805000115 30385636 PMC6243289

[22] Sturm A, Dworkin J. Phenotypic diversity as a mechanism to exit cellular dormancy. Current Biology. 2015;25(17):2272–2277. doi: 10.1016/j.cub.2015.07.018 26279233 PMC4778719

[23] Arnoldini M, Vizcarra IA, Peña-Miller R, Stocker N, Diard M, Vogel V, et al. Bistable expression of virulence genes in Salmonella leads to the formation of an antibiotic-tolerant subpopulation. PLOS biology. 2014;12(8):e1001928. doi: 10.1371/journal.pbio.1001928 25136970 PMC4138020

[24] Nikolic N, Schreiber F, Dal Co A, Kiviet DJ, Bergmiller T, Littmann S, et al. Cell-to-cell variation and specialization in sugar metabolism in clonal bacterial populations. PLOS genetics. 2017;13(12):e1007122. doi: 10.1371/journal.pgen.1007122 29253903 PMC5773225

[25] Shaffer SM, Dunagin MC, Torborg SR, Torre EA, Emert B, Krepler C, et al. Rare cell variability and drug-induced reprogramming as a mode of cancer drug resistance. Nature. 2017;546:431–435. doi: 10.1038/nature22794 28607484 PMC5542814

[26] Balaban NQ, Merrin J, Chait R, Kowalik L, Leibler S. Bacterial persistence as a phenotypic switch. Science. 2004;305:1622–1625. doi: 10.1126/science.1099390 15308767

[27] Nicolas D, Zoller B, Suter DM, Naef F. Modulation of transcriptional burst frequency by histone acetylation. Proceedings of the National Academy of Sciences. 2018;115:7153–7158. doi: 10.1073/pnas.1722330115 29915087 PMC6142243

[28] Symmons O, Chang M, Mellis IA, Kalish JM, Park J, Susztak K, et al. Allele-specific RNA imaging shows that allelic imbalances can arise in tissues through transcriptional bursting. PLOS Genetics. 2019;15:e1007874. doi: 10.1371/journal.pgen.1007874 30625149 PMC6342324

[29] Raj A, Peskin CS, Tranchina D, Vargas DY, Tyagi S. Stochastic mRNA synthesis in mammalian cells. PLOS Biology. 2006;4:e309. doi: 10.1371/journal.pbio.0040309 17048983 PMC1563489

[30] Chong S, Chen C, Ge H, Xie XS. Mechanism of Transcriptional Bursting in Bacteria. Cell. 2014;158:314–326. doi: 10.1016/j.cell.2014.05.038 25036631 PMC4105854

[31] Suter DM, Molina N, Gatfield D, Schneider K, Schibler U, Naef F. Mammalian genes are transcribed with widely different bursting kinetics. Science. 2011;332:472–474. doi: 10.1126/science.1198817 21415320

[32] Padovan-Merhar O, Nair GP, Biaesch AG, Mayer A, Scarfone S, Foley SW, et al. Single Mammalian Cells Compensate for Differences in Cellular Volume and DNA Copy Number through Independent Global Transcriptional Mechanisms. Molecular Cell. 2015;58:339–352. doi: 10.1016/j.molcel.2015.03.005 25866248 PMC4402149

[33] Vargas-Garcia CA, Ghusinga KR, Singh A. Cell size control and gene expression homeostasis in single-cells. Current opinion in systems biology. 2018;9:109–116. doi: 10.1016/j.coisb.2018.01.002 29862376 PMC5978733

[34] Soltani M, Singh A. Effects of Cell-Cycle-Dependent Expression on Random Fluctuations in Protein Levels. Royal Society Open Science. 2016;3:160578. doi: 10.1098/rsos.160578 28083102 PMC5210684

[35] Mena A, Medina DA, García-Martínez J, Begley V, Singh A, Chávez S, et al. Asymmetric cell division requires specific mechanisms for adjusting global transcription. Nucleic Acids Research. 2017;45:12401–12412. doi: 10.1093/nar/gkx974 29069448 PMC5716168

[36] Becskei A, Serrano L. Engineering stability in gene networks by autoregulation. Nature. 2000;405:590–593. doi: 10.1038/35014651 10850721

[37] Dublanche Y, Michalodimitrakis K, Kümmerer N, Foglierini M, Serrano L. Noise in transcription negative feedback loops: simulation and experimental analysis. Molecular Systems Biology. 2006;2:41. doi: 10.1038/msb4100081 16883354 PMC1681513

[38] Singh A, Hespanha JP. Optimal Feedback Strength for Noise Suppression in Autoregulatory Gene Networks. Biophysical Journal. 2009;96:4013–4023. doi: 10.1016/j.bpj.2009.02.064 19450473 PMC2712194

[39] Orrell D, Bolouri H. Control of internal and external noise in genetic regulatory networks. Journal of Theoretical Biology. 2004;230:301–312. doi: 10.1016/j.jtbi.2004.05.013 15302540

[40] Borri A, Palumbo P, Singh A. The impact of negative feedback in metabolic noise propagation. IET Systems Biology. 2016; p. 179–186. doi: 10.1049/iet-syb.2016.0003 27762232 PMC8687250

[41] Tao Y, Zheng X, Sun Y. Effect of feedback regulation on stochastic gene expression. Journal of Theoretical Biology. 2007;247:827–836. doi: 10.1016/j.jtbi.2007.03.024 17507034

[42] Kumar S, Lopez AJ. Negative feedback regulation among SR splicing factors encoded by Rbp1 and Rbp1-like in Drosophila. The EMBO Journal. 2005;24:2646–2655. doi: 10.1038/sj.emboj.7600723 15961996 PMC1176452

[43] Singh A, Hespanha JP. Evolution of autoregulation in the presence of noise. IET Systems Biology. 2009;3:368–378. 21028927 10.1049/iet-syb.2009.0002

[44] Stekel DJ, Jenkins DJ. Strong negative self regulation of Prokaryotic transcription factors increases the intrinsic noise of protein expression. BMC Systems Biology. 2008;2:6. doi: 10.1186/1752-0509-2-6 18205926 PMC2263017

[45] El-Samad H, Khammash M. Regulated Degradation Is a Mechanism for Suppressing Stochastic Fluctuations in Gene Regulatory Networks. Biophysical Journal. 2006;90:3749–3761. doi: 10.1529/biophysj.105.060491 16500958 PMC1440756

[46] Swain PS. Efficient Attenuation of Stochasticity in Gene Expression Through Post-transcriptional Control. Journal of Molecular Biology. 2004;344:956–976. doi: 10.1016/j.jmb.2004.09.073 15544806

[47] Voliotis M, Bowsher CG. The magnitude and colour of noise in genetic negative feedback systems. Nucleic Acids Research. 2012:7084–7095;. doi: 10.1093/nar/gks385 22581772 PMC3424552

[48] Alon U. Network motifs: theory and experimental approaches. Nature Reviews Genetics. 2007;8:450–461. doi: 10.1038/nrg2102 17510665

[49] Sawlekar R, Montefusco F, Kulkarni VV, Bates DG. Implementing Nonlinear Feedback Controllers Using DNA Strand Displacement Reactions. IEEE Transactions. 2016;15:443–454. 10.1109/TNB.2016.2560764 27164599

[50] Milias-Argeitis A, Summers S, Stewart-Ornstein J, Zuleta I, Pincus D, El-Samad H, et al. In silico feedback for in vivo regulation of a gene expression circuit. Nature Biotechnology. 2011;29:1114–1116. doi: 10.1038/nbt.2018 22057053 PMC4565053

[51] Buzi G, Khammash M. Implementation Considerations, Not Topological Differences, Are the Main Determinants of Noise Suppression Properties in Feedback and Incoherent Feedforward Circuits. PLOS Computational Biology. 2016;12:e1004958. doi: 10.1371/journal.pcbi.1004958 27257684 PMC4892630

[52] Uhlendorf J, Miermont A, Delaveau T, Charvin G, Fages F, Bottani S, et al. Long-term model predictive control of gene expression at the population and single-cell levels. IET Systems Biology. 2012;109:14271–14276. 10.1073/pnas.1206810109 PMC3435223 22893687

[53] Briat C, Gupta A, Khammash M. Antithetic Integral Feedback Ensures Robust Perfect Adaptation in Noisy Biomolecular Networks. Cell Systems. 2016;2:15–26. doi: 10.1016/j.cels.2016.02.010 27136686

[54] Qian Y, Vecchio DD. Realizing ‘integral control’ in living cells: how to overcome leaky integration due to dilution? Journal of The Royal Society Interface. 2018;15:20170902. doi: 10.1098/rsif.2017.0902 29436515 PMC5832733

[55] Wall ME, Hlavacek WS, Savageau MA. Design Principles for Regulator Gene Expression in a Repressible Gene Circuit. Journal of Molecular Biology. 2003;332:861–876. doi: 10.1016/S0022-2836(03)00948-3 12972257

[56] Aoki SK, Lillacci G, Gupta A, Baumschlager A, Schweingruber D, Khammash M. A universal biomolecular integral feedback controller for robust perfect adaptation. Nature. 2019;570:533–537. doi: 10.1038/s41586-019-1321-1 31217585

[57] Agrawal DK, Marshall R, Noireaux V, Sontag ED. In vitro implementation of robust gene regulation in a synthetic biomolecular integral controller perfect adaptation. Nature Communications. 2019;10:5760. doi: 10.1038/s41467-019-13626-z 31848346 PMC6917713

[58] El-Samad H, Kurata H, Doyle JC, Gross CA, Khammash M. Surviving heat shock: Control strategies for robustness and performance. Proceedings of the National Academy of Sciences. 2005;102(8):2736–2741. doi: 10.1073/pnas.0403510102 15668395 PMC549435

[59] Krakowiak J, Zheng X, Patel N, Feder ZA, Anandhakumar J, Valerius K, et al. Hsf1 and Hsp70 constitute a two-component feedback loop that regulates the yeast heat shock response. eLife. 2018;7:e31668. doi: 10.7554/eLife.31668 29393852 PMC5809143

[60] Sartori P, Tu Y. Noise Filtering Strategies in Adaptive Biochemical Signaling Networks. Journal of Statistical Physics. 2011;142:1206–1217. doi: 10.1007/s10955-011-0169-z 22977289 PMC3439208

[61] Yi TM, Huang Y, Simon MI, Doyle J. Robust perfect adaptation in bacterial chemotaxis through integral feedback control. Proceedings of the National Academy of Sciences. 2000;97:4649–4653. doi: 10.1073/pnas.97.9.4649 10781070 PMC18287

[62] Chevalier M, Gomez-Schiavon M, Ng A, El-Samad H. Design and analysis of a Proportional-Integral-Derivative controller with biological molecules. Cell Systems. 2019;9:338–353.e10. doi: 10.1016/j.cels.2019.08.010 31563473

[63] Van Kampen N. Stochastic Processes in Physics and Chemistry. Elsevier; 2011.

[64] Modi S, Soltani M, Singh A. Linear Noise Approximation for a Class of Piecewise Deterministic Markov Processes. 2018 Annual American Control Conference (ACC), Milwaukee, WI. 2018;1993–1998.

[65] Halpern KB, Tanami S, Landen S, Chapal M, Szlak L, Hutzler A, et al. Bursty Gene Expression in the Intact Mammalian Liver. Molecular Cell. 2015;58:147–156. doi: 10.1016/j.molcel.2015.01.027 25728770 PMC4500162

[66] Corrigan AM, Tunnacliffe E, Cannon D, Chubb JR. A continuum model of transcriptional bursting. eLife. 2016;5:e13051. doi: 10.7554/eLife.13051 26896676 PMC4850746

[67] Dar RD, Razooky BS, Singh A, Trimeloni TV, McCollum JM, Cox CD, et al. Transcriptional burst frequency and burst size are equally modulated across the human genome. Proceedings of the National Academy of Sciences. 2012;109:17454–17459. doi: 10.1073/pnas.1213530109 23064634 PMC3491463

[68] Singh A, Razooky BS, Dar RD, Weinberger LS. Dynamics of protein noise can distinguish between alternate sources of gene-expression variability. Molecular Systems Biology. 2012;8:607. doi: 10.1038/msb.2012.38 22929617 PMC3435505

[69] Fukaya T, Lim B, Levine M. Enhancer Control of Transcriptional Bursting. Cell. 2015;166:358–368. doi: 10.1016/j.cell.2016.05.025 PMC4970759 27293191

[70] Friedman N, Cai L, Xie XS. Linking stochastic dynamics to population distribution: an analytical framework of gene expression. Physical Review Letters. 2006;97:168302. doi: 10.1103/PhysRevLett.97.168302 17155441

[71] Shahrezaei V, Swain PS. Analytical distributions for stochastic gene expression. Proceedings of the National Academy of Sciences. 2008;105:17256–17261. doi: 10.1073/pnas.0803850105 18988743 PMC2582303

[72] Kumar N, Singh A, Kulkarni RV. Transcriptional Bursting in Gene Expression: Analytical Results for Genera Stochastic Models. PLOS Computational Biology. 2015;11:e1004292. doi: 10.1371/journal.pcbi.1004292 26474290 PMC4608583

[73] Kumar N, Platini T, Kulkarni RV. Exact Distributions for Stochastic Gene Expression Models with Bursting and Feedback. Physical Review Letters. 2014;113:268105. doi: 10.1103/PhysRevLett.113.268105 25615392

[74] Ghusinga KR, Dennehy JJ, Singh A. First-passage time approach to controlling noise in the timing of intracellular events. Proceedings of the National Academy of Sciences. 2017;114:693–698. doi: 10.1073/pnas.1609012114 28069947 PMC5278449

[75] Ghusinga KR, Singh A. Effect of gene-expression bursts on stochastic timing of cellular events. 2017 American Control Conference (ACC), Seattle, WA. 2017;2118–2123.

[76] Soltani M, Vargas C, Antunes D, Singh A. Intercellular Variability in Protein Levels from Stochastic Expression and Noisy Cell Cycle Processes. PLOS Computational Biology. 2016;12(8):e1004972. doi: 10.1371/journal.pcbi.1004972 27536771 PMC4990281

[77] Hespanha JP, Singh A. Stochastic Models for Chemically reacting Systems Using Polynomial Stochastic Hybrid Systems. International Journal of Robust and Nonlinear Control. 2005;15:669–689. doi: 10.1002/rnc.1017

[78] Singh A, Hespanha JP. Approximate Moment Dynamics for Chemically Reacting Systems. IEEE Transactions on Automatic Control. 2011;56:414–418. doi: 10.1109/TAC.2010.2088631

[79] Singh A. Negative feedback through mRNA provides the best control of gene-expression noise. IEEE transactions on nanobioscience. 2011;10:194–200. doi: 10.1109/TNB.2011.2168826 22020106

[80] Singh A, Hespanha JP. Stochastic hybrid systems for studying biochemical processes. Philosophical Transactions of the Royal Society A. 2010;368:4995–5011. doi: 10.1098/rsta.2010.0211 20921008

[81] Yu J, Xiao J, Ren X, Lao K, Xie XS. Probing Gene Expression in Live Cells, One Protein Molecule at a Time. Science. 2006;311:1600–1603. doi: 10.1126/science.1119623 16543458

[82] Singh A, Razooky B, Cox CD, Simpson ML, Weinberger LS. Transcriptional Bursting from the HIV-1 Promoter Is a Significant Source of Stochastic Noise in HIV-1 Gene Expression. Biophysical Journal. 2010;98:L32–L34. doi: 10.1016/j.bpj.2010.03.001 20409455 PMC2856162

[83] Ozbudak EM, Thattai M, Kurtser I, Grossman AD, van Oudenaarden A. Regulation of noise in the expression of a single gene. Nature Genetics. 2002;31:69–73. doi: 10.1038/ng869 11967532

[84] Dar RD, Shaffer SM, Singh A, Razooky BS, Simpson ML, Raj A, et al. Transcriptional bursting explains the noise–versus–mean relationship in mRNA and protein levels. PLOS ONE. 2016;11:e0158298. doi: 10.1371/journal.pone.0158298 27467384 PMC4965078

[85] Skupsky R, Burnett JC, Foley JE, Schaffer DV, Arkin AP. HIV Promoter Integration Site Primarily Modulates Transcriptional Burst Size Rather Than Frequency. PLOS Computational Biology. 2010;6:e1000952. doi: 10.1371/journal.pcbi.1000952 20941390 PMC2947985

[86] Singh A. Transient Changes in Intercellular Protein Variability Identify Sources of Noise in Gene Expression. Biophysical Journal. 2014;107:2214–2220. doi: 10.1016/j.bpj.2014.09.017 25418106 PMC4223191

[87] Roberts E, Be’er S, Bohrer C, Sharma R, Assaf M. Dynamics of simple gene-network motifs subject to extrinsic fluctuations. Phys. Rev. E. 2015;92:062717. doi: 10.1103/PhysRevE.92.062717 26764737

[88] Modi S, Singh A. Controlling organism size by regulating constituent cell numbers. 2018 IEEE Conference on Decision and Control (CDC), Miami, FL. 2018;2685–2690. 10.1109/CDC.2018.8619546 PMC6420813 30886453

[89] Dar RD, Razooky BS, Singh A, Trimeloni TV, McCollum JM, Cox CD, Simpson ML, Weinberger LS. Transcriptional burst frequency and burst size are equally modulated across the human genome. Proceedings of the National Academy of Sciences. 2012;17454–17459. doi: 10.1073/pnas.1213530109 23064634 PMC3491463

[90] Singh A, Soltani M. Quantifying Intrinsic and Extrinsic Variability in Stochastic Gene Expression Models. PLOS ONE. 2013;8:e84301. doi: 10.1371/journal.pone.0084301 24391934 PMC3877255

[91] Paulsson J. Model of stochastic gene expression. Physics of Life Reviews. 2005;2:157–175. doi: 10.1016/j.plrev.2005.03.003

[92] Hilfinger A, Paulsson J. Separating intrinsic from extrinsic fluctuations in dynamic biological systems. Proceedings of the National Academy of Sciences. 2011;108:12167–12172. doi: 10.1073/pnas.1018832108 21730172 PMC3141918

[93] Swain PS, Elowitz MB, Siggia ED. Intrinsic and extrinsic contributions to stochasticity in gene expression. Proceedings of the National Academy of Sciences. 2002;99:12795–12800. doi: 10.1073/pnas.162041399 12237400 PMC130539

[94] Paulsson J. Summing up the noise in gene networks. 2004;427:415–418. 10.1038/nature02257 14749823

[95] Alon U. An Introduction to Systems Biology: Design Principles of Biological Circuits. Chapman and Hall/CRC; 2011.

[96] Dey S, Soltani M, Singh A. Enhancement of gene expression noise from transcription factor binding to genomic decoy sites. Scientific Reports. 2020;10:9126. doi: 10.1038/s41598-020-65750-2 32499583 PMC7272470

[97] Lestas I, Vinnicombe G, Paulsson J. Fundamental limits on the suppression of molecular fluctuations. Nature. 2010;467:174–178. doi: 10.1038/nature09333 20829788 PMC2996232

[98] Briat C, Zechner C, Khammash M. Design of a Synthetic Integral Feedback Circuit: Dynamic Analysis and DNA Implementation. ACS Synthetic Biology. 2016;5:1108–1116. doi: 10.1021/acssynbio.6b00014 27345033

[99] Klavins E. Proportional-Integral Control of Stochastic Gene Regulatory Networks. 49th IEEE Conference on Decision and Control (CDC), Atlanta, GA. 2010;2547–2553.

[100] Pérez-Ortín JE, Mena A, Barba-Aliaga M, Alonso-Monge R, Singh A, Chávez S, et al. Cell volume homeostatically controls the rDNA repeat copy number and rRNA synthesis rate in yeast. PLOS Genetics. 2021;17:1–18. doi: 10.1371/journal.pgen.1009520 33826644 PMC8055003

[101] Rosenfeld N, Elowitz MB, Alon U. Negative autoregulation speeds the response times of transcription networks. Journal of Molecular Biology. 2002;323:785–793. doi: 10.1016/S0022-2836(02)00994-4 12417193

[102] Gillespie DT. A General Method for Numerically Simulating the Stochastic Time Evolution of Coupled Chemical Reactions. Journal of Computational Physics. 1976;22:403–434. doi: 10.1016/0021-9991(76)90041-3

[103] Thieffry D, Huerta AM, PÃ©rez-Rueda E, Collado-Vides J. From specific gene regulation to genomic networks: a global analysis of transcriptional regulation in Escherichia coli. Bioessays. 1998;20:433–440. doi: 10.1002/(SICI)1521-1878(199805)20:5<433::AID-BIES10>3.0.CO;2-2 9670816

[104] Hornung G, Barkai N. Noise Propagation and Signaling Sensitivity in Biological Networks: A Role for Positive Feedback. PLOS Computational Biology. 2008;4:1–7. doi: 10.1371/journal.pcbi.0040008 18179281 PMC2174979

[105] Mangan S, Alon U. Structure and function of the feed-forward loop network motif. Proceedings of the National Academy of Sciences. 2003;11980–11985. doi: 10.1073/pnas.2133841100 14530388 PMC218699

[106] Hong J, Brandt N, Abdul-Rahman F, Yang A, Hughes T, Gresham D. An incoherent feedforward loop facilitates adaptive tuning of gene expression. eLife. 2018;7:e32323. doi: 10.7554/eLife.32323 29620523 PMC5903863

[107] Joanito I, Chu J, Wu S, Hsu C. An incoherent feed-forward loop switches the Arabidopsis clock rapidly between two hysteretic states. Scientific Reports. 2018;8:13944. doi: 10.1038/s41598-018-32030-z 30224713 PMC6141573

[108] Yoshida T, Matsuda M, Hirashima T. Incoherent Feedforward Regulation via Sox9 and ERK Underpins Mouse Tracheal Cartilage Development. Frontiers in Cell and Developmental Biology. 2020;8:1075. doi: 10.3389/fcell.2020.585640 33195234 PMC7642454

[109] Whitby M, Cardelli L, Kwiatkowska M, Laurenti L, Tribastone M, Tschaikowski M. PID Control of Biochemical Reaction Networks. 58th IEEE Conference on Decision and Control (CDC), Nice, France. 2019;8372–8379.

[110] Vahdat Z, Nienaltowski K, Farooq Z, Komorowski M, Singh A. Information processing in unregulated and autoregulated gene expression. 2020 European Control Conference (ECC), Saint Petersburg, Russia. 2020;258–263.

