# Supplementary material for: Noise suppression in stochastic genetic circuits using PID controllers
Source: PLoS Comput Biol. 2021 Jul 28;17(7):e1009249. doi: 10.1371/journal.pcbi.1009249 (PMC8360635; doi:10.1371/journal.pcbi.1009249)
Supplement: S1 Text — In addition, Fig A shows the analytical vs simulation results for the proportional controller, Fig B shows the analytical vs simulation results for integral controller, and Fig C shows the simulation results for the combined circuit of proportional and integral controller. Fig D shows the effect of changing the activation of Z by Y on total noise. (PDF) [file pcbi.1009249.s001.pdf]

Supplementary Information for "Noise suppression in stochastic genetic circuits using PID controllers"

Saurabh Modi, Supravat Dey, and Abhyudai Singh

I. Comparison of analytical results with stochastic simulations

We perform stochastic simulations for the genetic circuits with different controllers presented in the main paper, using the Gillespie algorithm [1]. The analytical results for the target protein (Y) noise obtained using the linear noise approximation are compared with the numerical simulations. For this, we compute the noise in the target protein (Y) at the steady-state (from a large number of samples, $\sim 10^6$) as a function of the feedback gain, keeping the mean levels constant.

Strategy for changing feedback gain maintaining constant mean levels

For the proportional and integral controller implementations, the repression from the sensor (Z) to the target protein Y is incorporated via a Hill function,

$$g(z) = \frac{1}{1 + (z/z_c)^h}, \tag{S1}$$

where h is the Hill coefficient and z_c is the amount of Z at which half-maximal repression is achieved. Then, the feedback gain given by Eq. (25) and Eq. (40) (main manuscript) reduces to

$$f_p, f_i := -\frac{\overline{\langle z \rangle}}{g(\overline{\langle z \rangle})} \frac{dg(z)}{dz}\bigg|_{z=\overline{\langle z \rangle}} = h(1 - g(\overline{\langle z \rangle})). \tag{S2}$$

To increase feedback gain, we vary h value. The simulation results presented below are obtained by choosing $z_c = \overline{\langle z \rangle}$, where $g(\overline{\langle z \rangle}) = 1/2$ and $f_p, f_i = h/2$. As $g(\overline{\langle z \rangle})$ is independent of h, it follows from Eq. (21), (23) and (41) in the main manuscript, that the steady state mean levels are independent of the feedback gain.

Simulation results for the proportional controller

For all the simulation results presented in this SI, we consider shifted geometric distributions for the production of species:

$$\mathbb{P}(B = j) = \frac{[1 - 1/\langle B \rangle]^{j-1}}{\langle B \rangle} \text{ for } j \in \{1, 2, 3, ...\}, \tag{S3}$$

where, $\langle B \rangle$ is the average burst size. In Fig A, we compare analytical results with corresponding estimations from stochastic simulations. The simulations results show good quantitative agreement with their analytical estimate for smaller values of the sensor noise. For a high sensor noise, a deviation is observed for larger values of the feedback gain. However, the qualitative behavior remains the same as the analytical prediction. We note that our analytical calculation is based

on the assumption that the fluctuations around the mean copy numbers are relatively small. As a result, the linear noise approximation breaks down when in the presence of large fluctuations and strong nonlinearity.

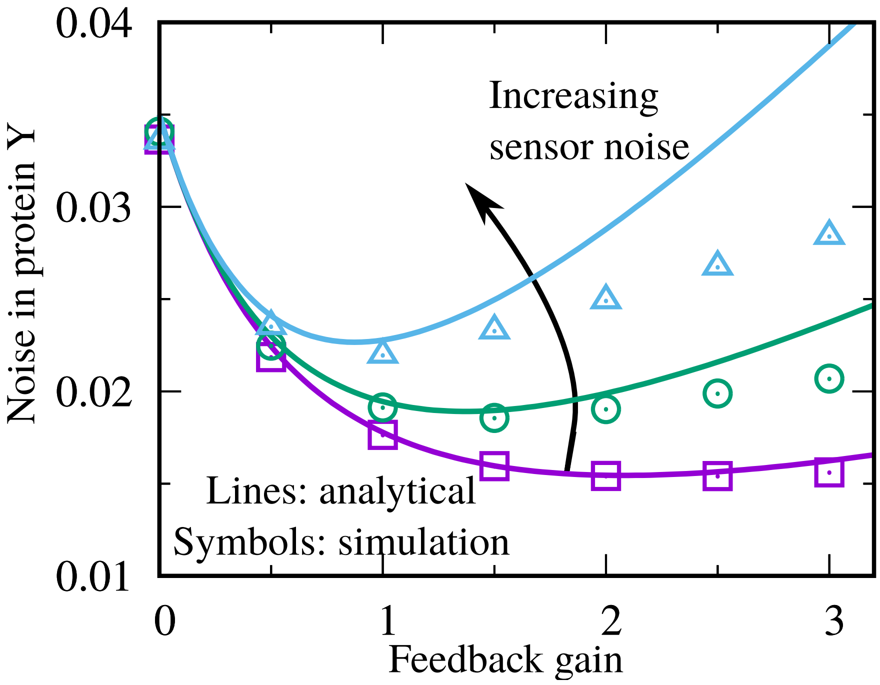

Fig A: **Analytical vs simulation results for the proportional controller:** The noise in Y copy number (CV_Y^2) is plotted against the feedback gain for different values of the sensor noise ($CV_Z^2 = 0.02,\ 0.04$, and 0.08). The lines represent analytical estimates and symbols stand for corresponding results from stochastic simulations. The simulations results show good agreement with their analytical estimate for a small sensor noise. For a high sensor noise, the quantitative agreement is poor for higher feedback gains. For this plot, the steady-state mean values of copy numbers in X, Y, and Z are kept fixed to $\overline{\langle x \rangle} = 100$, $\overline{\langle y \rangle} = 400$, and $\overline{\langle z \rangle} = 100$. Feedback gain is changed by varying h. Other parameter values are: degradation rates – $\gamma_x = 1.0$, $\gamma_y = 3.0$, $\gamma_z = 15.0$; average burst sizes – $\langle B_x \rangle = 2$, and $\langle B_y \rangle = 8$. We vary $\langle B_z \rangle$ to increase the sensor noise.

Implementation of zeroth order decay for the integral controller circuit

For this, we assume the Michaelis-Menten decay kinetics [2] for the sensor protein

$$\mathbb{P}(z(t+dt) = z(t) - 1 | z(t)) = \frac{v_{max} z}{z + k_M} dt, \tag{S4}$$

where v_{max} is the maximum decay rate achieved by the enzyme-substrate interaction and k_M is the amount of Z needed to attain half of the maximum decay rate. In the limit of $k_M \ll z$, the decay follows the zeroth-order kinetics. In this limit, the deterministic equation for the Z dynamics satisfy Eq. (36) (main manuscript) for the following choice of v_{max} and k_M:

$$v_{max} = \left(1 + \frac{k_M}{\overline{\langle z \rangle}}\right) k_z \overline{\langle y \rangle}, \text{ with } \frac{k_M}{\overline{\langle z \rangle}} \ll 1. \tag{S5}$$

Simulation results for the integral controller

In Fig B, we compare analytical and simulation results for the integral controller. The quantitative agreement between them is good for smaller values of the sensor noise. As in the case of the proportional controller, a deviation is observed for a high sensor noise with larger values of the feedback gain. The qualitative behavior, however, follows to the analytical prediction. This deviation is attributed to the linearization approximation on the basis small noise assumption which violates in the presence of large fluctuations and strong nonlinearity.

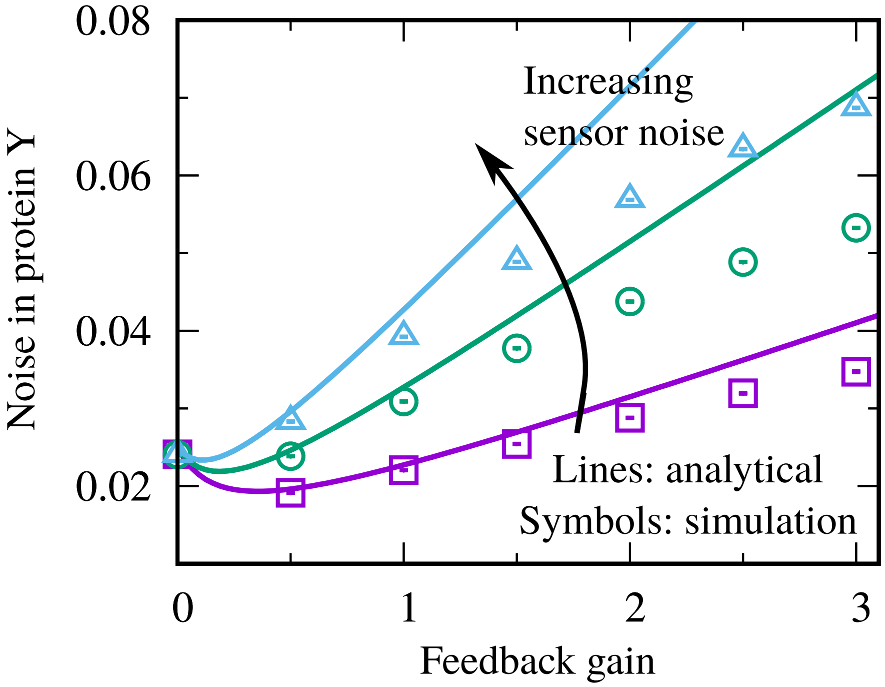

Fig B: **Analytical vs simulation results for integral controller:** The noise in Y copy number (CV_Y^2) is plotted against the feedback gain for different values of the sensor noise ($CV_Z^2 = 0.01,\ 0.02,$ and 0.03). The lines represent analytical estimates and symbols stand for corresponding results from stochastic simulations. The simulations results show good agreement with their analytical estimate for a small sensor noise. For a high sensor noise, the quantitative agreement is poor for higher feedback gains. For this plot, the steady-state mean values of copy numbers in X, Y, and Z are kept fixed to $\overline{\langle x \rangle} = 100$, $\overline{\langle y \rangle} = 200$, and $\overline{\langle z \rangle} = 100$. Feedback gain is changed by varying h. Other parameter values are: $\gamma_x = 1.0$, $\gamma_y = 3.0$, $k_z = \gamma_y$, $\langle B_x \rangle = \langle B_y \rangle = 2$. We vary $\langle B_z \rangle$ to increase the sensor noise.

II. A combined circuit of proportional and integral controllers

In the main paper, we consider the proportional, integral, and derivative controller separately. How does a combined PI (proportional and integral) implementation work in terms of target protein noise as opposed to the individual controllers? To study this, we consider a combined PI system in the presence of extrinsic noise and solve numerically. The circuit is schematically shown in Fig C(i). This system consists of four species X (for extrinsic noise), Y (target protein), Z_p (proportional controller), and Z_i (integral controller). The species Y activates both controllers linearly. Both controller species Z_p and Z_i repress the synthesis of the target protein Y, and this repression is

modelled the following two-dimensional function,

$$g_{pi}(z_p, z_i) = \frac{1}{1 + (z_p/z_c)^{h_p} + (z_i/z_c)^{h_i}}, \tag{S6}$$

where h_p and h_i are the Hill coefficients and z_c is the value of repression coefficient which we assume to be the same for both the species for simplicity [3]. The dynamics of external noise is implemented in the same way as described in the main text. The stochastic dynamics of Z_p, Z_i, and Y are given by,

$$\mathbb{P}(z_p(t + dt) = z_p(t) + j|y(t), z_p(t), z_i(t)) = k_{z_p} y \mathbb{P}(B_z = j)dt, \tag{S7a}$$

$$\mathbb{P}(z_p(t + dt) = z_p(t) - 1|y(t), z_p(t), z_i(t)) = \gamma_{z_p} z_p dt, \tag{S7b}$$

$$\mathbb{P}(z_i(t + dt) = z_i(t) + j|y(t), z_p(t), z_i(t)) = k_{z_i} y \mathbb{P}(B_z = j)dt, \tag{S7c}$$

$$\mathbb{P}(z_i(t + dt) = z_i(t) - 1|y(t), z_p(t), z_i(t)) = \frac{v_{max} z_i}{z_i + k_M} dt, \tag{S7d}$$

$$\mathbb{P}(y(t + dt) = y(t) + j|y(t), x(t), z_p(t), z_i(t)) = \frac{k_y g_{ip}(z_p, z_i) x}{\overline{\langle x \rangle}} \mathbb{P}(B_y = j)dt, \tag{S7e}$$

$$\mathbb{P}(y(t + dt) = y(t) - 1|y(t), x(t), z_p(t), z_i(t)) = \gamma_y y dt. \tag{S7f}$$

Note that the zero-order decay of Z_i is implemented using Michaelis-Menten decay kinetics as described above (Eqs. S4 and S5). For simplicity, we choose the burst size distributions for both controllers identical and independent. For the combined repression function (Eq. S6), the proportional and integral feedback gain can be defined as,

$$f_p := -\frac{\overline{\langle z_p \rangle}}{g_{ip}(\overline{\langle z_p \rangle}, \overline{\langle z_i \rangle})} \frac{\partial g_{ip}(z_p, z_i)}{\partial z_p}\bigg|_{z_p = \overline{\langle z_p \rangle}, z_i = \overline{\langle z_i \rangle}} = h_p(1 - 2g_{ip}(\overline{\langle z_p \rangle}, \overline{\langle z_i \rangle})), \tag{S8}$$

$$f_i := -\frac{\overline{\langle z_p \rangle}}{g_{ip}(\overline{\langle z_p \rangle}, \overline{\langle z_i \rangle})} \frac{\partial g_{ip}(z_p, z_i)}{\partial z_i}\bigg|_{z_p = \overline{\langle z_p \rangle}, z_i = \overline{\langle z_i \rangle}} = h_i(1 - 2g_{ip}(\overline{\langle z_p \rangle}, \overline{\langle z_i \rangle})). \tag{S9}$$

To increase feedback gains, we vary the value of Hill coefficients h_p and h_i. The simulation results presented below are obtained by choosing $z_c = \overline{\langle z_p \rangle} = \overline{\langle z_i \rangle}$, where $g_{ip}(\overline{\langle z_p \rangle}, \overline{\langle z_i \rangle}) = 1/3$, $f_p = h_p/3$, and $f_i = h_i/3$.

We simulate the stochastic dynamics of X, Y, Z_p, and Z_i using Gillespie algorithm and compute the noise in the target proteins at the steady-state from a large number of sample points. In Fig C, we plot the target protein noise (normalized by target protein noise for open loop circuit) as a function of the proportional feedback gain f_p and integral feedback gain f_i. When the extrinsic noise is small, the only proportional controller case ($f_i = 0$ in Fig C) is the best for noise reduction in Y. Interestingly, when extrinsic noise is large, the combined PI controller performs better as opposed to individual controllers ($f_i = 0$ or $f_p = 0$ axes).

III. Parametric constraints for derivative control

The burst frequency function for target protein synthesis, which depends on both the target protein (Y) and controller species (Z), must obey particular constraints to act as a derivative controller. Here we discuss this limitation. Let $g(y, z)$ be the general burst frequency function. We can linearize it around the mean as

$$g(y, z) - g\left(\overline{\langle y \rangle}, \overline{\langle z \rangle}\right) \approx g\left(\overline{\langle y \rangle}, \overline{\langle z \rangle}\right) \left(S_z \frac{z}{\overline{\langle z \rangle}} + S_y \frac{y}{\overline{\langle y \rangle}}\right). \tag{S10}$$

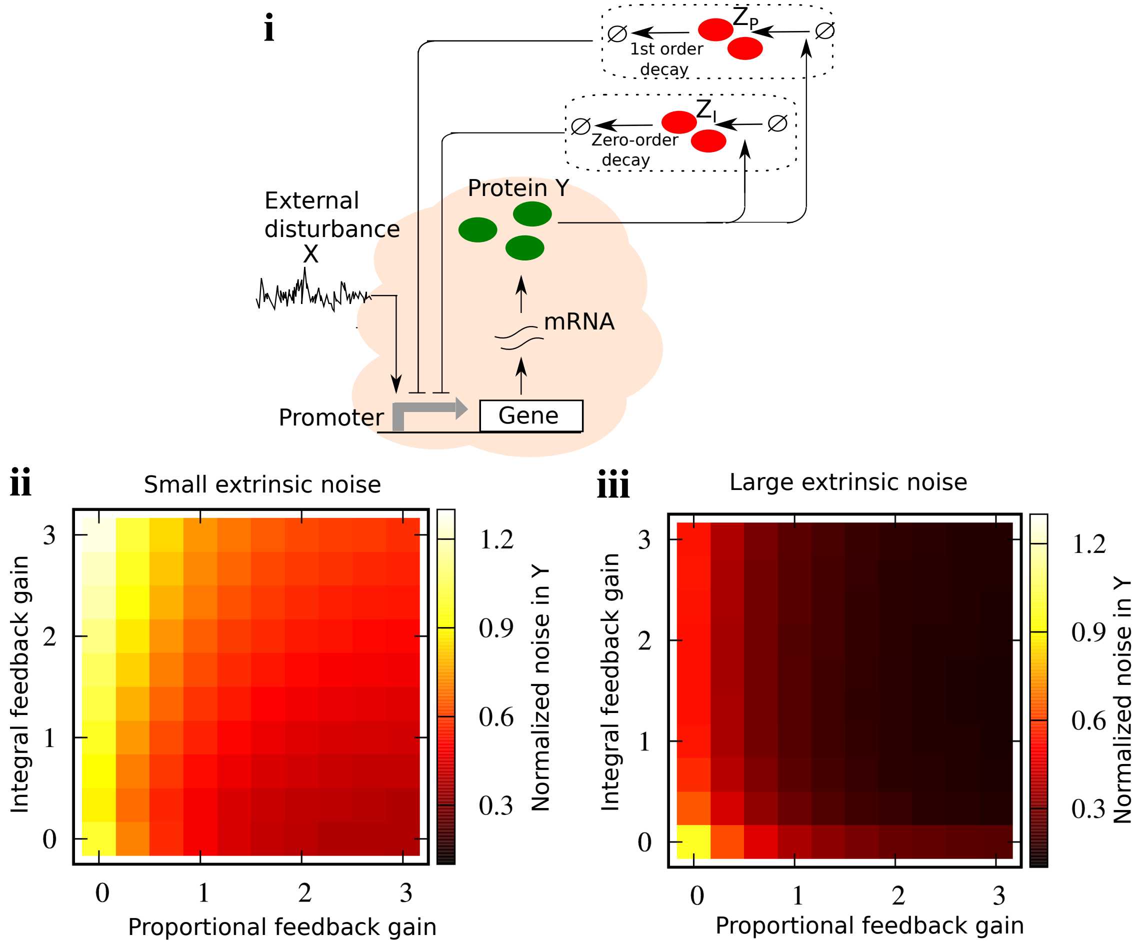

Fig C: **Simulation results for the combined circuit of proportional and integral controller.** (i) Schematic diagram of the circuit. (ii) Density plot for normalized noise in Y (normalized by corresponding noise for open loop circuit) as a function of both feedback gains when extrinsic noise is small ($CV_x^2 = 0.02$). In this case, individual proportional controller (for $f_i = 0$) works better than the combined circuit in terms of noise reduction. (iii) Density plot for normalized noise in Y when extrinsic noise is large ($CV_x^2 = 0.2$). The noise for combined PI ($f_p > 0$ and $f_i > 0$) case can be smaller than individual controllers ($f_i = 0$ or $f_p = 0$). Thus, in this case, a combination of P and I can be better for noise reduction as opposed to individual P and I controllers. For this figure, the steady-state mean values of copy numbers in X, Y, and Z are kept fixed to $\overline{\langle x \rangle} = 100$, $\overline{\langle y \rangle} = 100$, and $\overline{\langle z \rangle} = 100$. Feedback gains f_p and f_i are changed by varying h_p and h_i respectively. Other parameter values are: $\gamma_x = 1.0$, $\gamma_y = 3.0$, $\gamma_{z_p} = 15.0$, $k_{z_i} = \gamma_y$, $\langle B_y \rangle = 2$ and $\langle B_z \rangle = 1$.

Here, S_z and S_y are normalized sensitivities of $g(y,z)$ with respect to Z and Y respectively given by

$$S_y = \frac{\overline{\langle y \rangle}}{g(\overline{\langle y \rangle}, \overline{\langle z \rangle})} \frac{\partial g(y,z)}{\partial y}\bigg|_{y=\overline{\langle y \rangle}, z=\overline{\langle z \rangle}}, \text{ and } S_z = \frac{\overline{\langle z \rangle}}{g(\overline{\langle y \rangle}, \overline{\langle z \rangle})} \frac{\partial g(y,z)}{\partial z}\bigg|_{y=\overline{\langle y \rangle}, z=\overline{\langle z \rangle}}. \quad \text{(S11)}$$

Using Eq. (49) in the main text, and assuming γ_z is large (i.e., fast sensor dynamics), the Laplace transform of the right-hand-side of (S10) is

$$g\left(\overline{\langle y\rangle},\overline{\langle z\rangle}\right)\left(S_z\frac{Z(s)}{\overline{\langle z\rangle}}+S_y\frac{Y(s)}{\overline{\langle y\rangle}}\right)=g\left(\overline{\langle y\rangle},\overline{\langle z\rangle}\right)\left(\frac{S_z}{\overline{\langle y\rangle}}\frac{Y(s)}{\frac{s}{\gamma_z}+1}+S_y\frac{Y(s)}{\overline{\langle y\rangle}}\right) \tag{S12}$$

$$\approx\frac{g\left(\overline{\langle y\rangle},\overline{\langle z\rangle}\right)}{\overline{\langle y\rangle}}\left((S_z+S_y)Y(s)+\frac{S_y sY(s)}{\gamma_z}\right). \tag{S13}$$

Here, the first term on the RHS represents proportional control while the second term is corresponds to derivative control. Therefore, this represents combined proportional derivative controller. A pure derivative controller is obtained only when $S_z=-S_y$ for which the first term drops out of the equation.

For the regulation of burst frequency Y as shown in the main text, with Hill functions for the activation and repression we get Eq. (50) in the main text. Using $S_z=-S_y$ derived above and (S11) we get the following relationship

$$h_z\left(\frac{1}{1+(\overline{\langle z\rangle}/z_c)^{h_z}}\right)=h_y\left(1-\frac{1}{1+(\overline{\langle y\rangle}/y_c)^{h_y}}\right). \tag{S14}$$

This relationship simplifies to $h_z=h_y=h$ for $z_c\gg\overline{\langle z\rangle}$ and $y_c\ll\overline{\langle y\rangle}$ and leads to $g(y,z)=(z/y)^h$ as reported in the main text.

In this analysis we show that while the general derivative control requires $S_z=-S_y$, for simplicity we choose the formulation consisting of Hill functions with strong binding affinity of the regulators with the same Hill coefficients.

IV. Dependence of target protein noise on $Y\rightarrow Z$ activation strength

In the main text, we mostly discussed the results for a given activation strength of $Y\rightarrow Z$ reaction, i.e. for a fixed $\overline{\langle z\rangle}$ value. Here, we analyze how the target protein noise depends on the $\overline{\langle z\rangle}$.

In the proportional controller, we can change $\overline{\langle z\rangle}$ by varying k_z which represents the production rate of Z due to the activation by Y given by Eq. (21) in the main manuscript. This changes CV_Z^2 according to Eq. (31) and changes f_p based on Eq. (27). For a fair comparison of CV_Y^2 with changing k_z we need to keep $\overline{\langle y\rangle}$ constant. To keep $\overline{\langle y\rangle}$ constant, we vary the maximum transcription rate k_y following Eq. (23). With increasing $\overline{\langle z\rangle}$, the feedback strength increases according to

$$f_p=h\left(1-\frac{1}{1+(\overline{\langle z\rangle}/z_c)^h}\right). \tag{S15}$$

So, for low $\overline{\langle z\rangle}$, $f_p\propto h\overline{\langle z\rangle}^h$ and given that

$$CV_Z^2=\frac{\langle B_z\rangle+\langle B_z^2\rangle}{2\langle B_z\rangle\overline{\langle z\rangle}} \tag{S16}$$

the sensor noise component for low $\overline{\langle z\rangle}$ becomes

$$\overbrace{\frac{f_p^2\gamma_y}{(f_p+1)(\gamma_y+\gamma_z)}CV_Z^2}^{\text{Sensor noise}}\propto h^2\overline{\langle z\rangle}^{2h-1} \tag{S17}$$

which increases with $\overline{\langle z \rangle}$. However for high values, as $f_p \propto h\overline{\langle z \rangle}^0$, the sensor noise component $\propto h\overline{\langle z \rangle}^{-1}$ which is decreasing. This leads to a maxima for sensor noise at intermediate values as seen in Fig D (i,ii). Note that for higher h, this effect is amplified due to the presence of the scaling of the sensor noise component with respect to h. The other two components are monotonically buffered with increase in $\overline{\langle z \rangle}$ which reflects the sole increase in f_p in

$$\overbrace{\frac{(\gamma_y + f_p\gamma_y + \gamma_z)}{(f_p+1)(\gamma_y+\gamma_z)} CV_{int}^2}^{\text{Intrinsic noise}} + \overbrace{\frac{\gamma_y((\gamma_z+\gamma_y)(\gamma_x+\gamma_z)+\gamma_x\gamma_y f_p)}{(1+f_p)(\gamma_y+\gamma_z)((\gamma_x+\gamma_y)(\gamma_x+\gamma_z)+\gamma_y\gamma_z f_p)} CV_X^2}^{\text{External disturbance}} . \tag{S18}$$

For the integral controller, $\overline{\langle z \rangle}$ is a parameter which can be increased independent of k_z due to the way we have defined its dynamics given in Eq. (36) in the main manuscript. CV_Z^2 and f_i change in a similar fashion to those in proportional controller. We compensate with k_y to keep $\overline{\langle y \rangle}$ constant based on Eq. (41). The sensor noise in this case given by

$$\overbrace{f_i CV_{int}^2}^{\substack{\text{Intrinsic}\\ \text{noise}}} \propto h \left(\frac{\overline{\langle z \rangle}^{h-1}}{1+(\overline{\langle z \rangle}/z_c)^h} \right). \tag{S19}$$

This shows that for $h = 1$, the noise decreases, while for $h > 1$, it follows the same profile as in the proportional controller case as seen in Fig D (iii,iv). While the noise from stochastic expression is independent of f_i and CV_Z^2, the extrinsic noise follows

$$\overbrace{\frac{\overline{\langle z \rangle}\gamma_x\gamma_y}{(\overline{\langle y \rangle} f_i k_z \gamma_y + \overline{\langle z \rangle}(\gamma_y\gamma_x+\gamma_x^2))} CV_X^2}^{\text{External disturbance}} \tag{S20}$$

which is an increasing function with respect to $\overline{\langle z \rangle}$ for the regime where $h = 1$ (Fig D (iii)). In the highly non-linear regime $h = 6$, f_i increases steeply at intermediate values of $\overline{\langle z \rangle}$ and causes a slight U-shaped profile as seen in Fig D (iv).

For the derivative controller, we can change $\overline{\langle z \rangle}$ by varying k_z which represents the production rate of Z due to the activation by Y given by Eq. (48) in the main manuscript. To keep $\overline{\langle y \rangle}$ constant, we vary the maximum transcription rate k_y following Eq. (53). In this analysis, the feedback gain f_d is independent of $\overline{\langle z \rangle}$ and we find that CV_Y^2 follows the change in CV_Z^2. CV_Z^2 reduces asymptotically with increasing level of Z as seen in (S16). This gives the monotonically decreasing profile as seen in Fig D (v,vi).

V. External disturbance as an OU process

In this section we derive the expression for CV_Y^2 without feedback using the dynamics of X given by:

$$dx(t) = \gamma_x(\overline{\langle x \rangle} - x(t))dt + \sigma_x dw(t). \tag{S21}$$

Here, $\overline{\langle x \rangle}$ is the mean level of the external disturbance, γ_x is the time scale of the disturbance, σ_x represents strength of the disturbance and $w(t)$ is the Wiener process. The hybrid system is then given by the above OU process along with the dynamics of Y shown in Eq. (13) in the main text. The resulting moment dynamics for an arbitrary function $\phi(x,y)$ using an extended generator L of

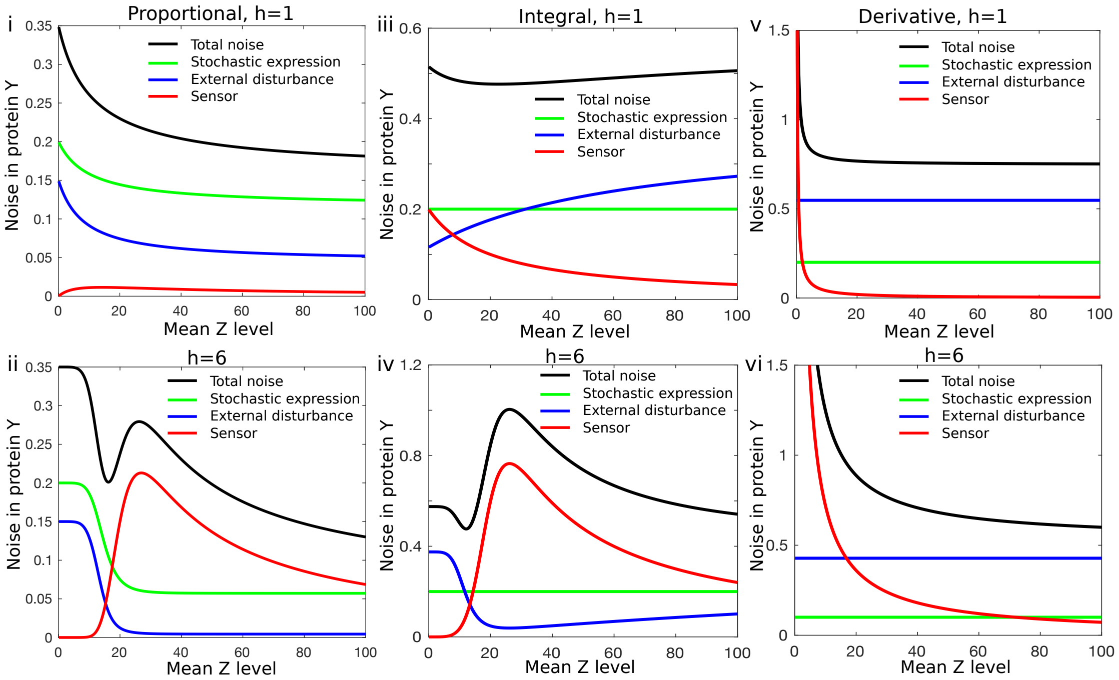

Fig D: **Effect of changing the activation of Z by Y on total noise.** (i) & (ii) The noise in Y for proportional controller with respect to $\overline{\langle z\rangle}$ for $h = 1$ and $h = 6$ respectively is shown. Parameters used are: $CV^2_{int} = CV^2_X = 0.2$, $\gamma_z = 5\gamma_y = 3\gamma_x$, $z_c = 20$, and $\langle B_z\rangle = 8$ with shifted geometrically distributed bursts. Noise in Y is obtained from Eq. (30). (iii) & (iv) The noise in Y for integral controller with respect to $\overline{\langle z\rangle}$ for $h = 1$ and $h = 6$ respectively is shown. Parameters used are: $CV^2_{int} = 0.2$, $CV^2_X = 0.5$, $\gamma_y = 3\gamma_x$, $k_z = \gamma_y$, $\overline{\langle y\rangle} = z_c = 20$, and $\langle B_z\rangle = 4$ with shifted geometrically distributed bursts. Noise in Y is obtained from Eq. (42). (v) & (vi) The noise in Y for the derivative controller with respect to $\overline{\langle z\rangle}$ for $h = 1$ and $h = 6$ respectively is shown. Parameters used are: $CV^2_{int} = 0.25$, $CV^2_X = 0.7$, $\gamma_z = 3\gamma_y = 5\gamma_x$, and $\langle B_z\rangle = 2$ with shifted geometrically distributed bursts. Noise in Y is obtained from Eq. (57).

stochastic hybrid system are given by [4]

$$\frac{\langle\phi(x,y)\rangle}{dt} = \langle (L\phi)(x,y)\rangle. \tag{S22}$$

The generator is given by

$$\begin{aligned}(L\phi)(x,y) :=& \gamma_x \frac{\partial\phi(x,y)}{\partial x}(\overline{\langle x\rangle} - x) + \frac{1}{2}\frac{\partial^2\phi(x,y)}{\partial x^2}\sigma_x^2 + \sum_{j=0}^{\infty} k_y \frac{x}{\overline{\langle x\rangle}}\mathbb{P}(B_y = j)(\phi(x,y+j) - \phi(x,y)) \\ &+ \gamma_y y(\phi(x,y-1) - \phi(x,y)).\end{aligned} \tag{S23}$$

The resulting second order moment equations are found by appropriately substituting for the function $\phi(x, y)$

$$\frac{\langle y^2 \rangle}{dt} = -2\gamma_y \langle y^2 \rangle + \gamma_y \langle y \rangle + k_y \langle B_y^2 \rangle + 2\langle B_y \rangle k_y \frac{\langle xy \rangle}{\overline{\langle x \rangle}} \tag{S24}$$

$$\frac{\langle xy \rangle}{dt} = \gamma_x(\langle x \rangle \langle y \rangle - \langle xy \rangle) + k_y \langle B_y \rangle \frac{\langle x^2 \rangle}{\overline{\langle x \rangle}} - \gamma_y \langle xy \rangle \tag{S25}$$

$$\frac{\langle x^2 \rangle}{dt} = \gamma_x(2\overline{\langle x \rangle}\langle x \rangle - 2\langle x^2 \rangle) + \sigma_x^2. \tag{S26}$$

Solving these equations and obtaining the expression for CV_Y^2 in terms of CV_X^2 we see that is identical to Eq. (17) in the main text

$$CV_Y^2 = \overbrace{CV_{int}^2}^{\substack{\text{Intrinsic} \\ \text{noise}}} + \overbrace{\frac{\gamma_y}{(\gamma_y + \gamma_x)} CV_X^2}^{\text{External disturbance}}, \tag{S27}$$

where $CV_X^2 = \frac{\sigma_x^2}{2\gamma_x \overline{\langle x \rangle}^2}$ instead of Eq. (12) in the main text.

Supporting References

[1] D. T. Gillespie. A general method for numerically simulating the stochastic time evolution of coupled chemical reactions. *Journal of Computational Physics*, 22:403–434, 1976.

[2] Leonor Michaelis and Maud Menten. Die kinetik der invertinwirkung. *Biochem. Z.*, 49:333–369, 1913.

[3] Uri Alon. *An Introduction to Systems Biology: Design Principles of Biological Circuits.* Chapman and Hall/CRC, 2011.

[4] Abhyudai Singh and J. P. Hespanha. Stochastic hybrid systems for studying biochemical processes. *Philosophical Transactions of the Royal Society A*, 368:4995–5011, 2010.
